# Long-Range Chromosome Interactions Mediated by Cohesin Shape Circadian Gene Expression

**DOI:** 10.1371/journal.pgen.1005992

**Published:** 2016-05-02

**Authors:** Yichi Xu, Weimin Guo, Ping Li, Yan Zhang, Meng Zhao, Zenghua Fan, Zhihu Zhao, Jun Yan

**Affiliations:** 1 CAS-MPG Partner Institute for Computational Biology, Shanghai Institutes for Biological Sciences, Chinese Academy of Sciences, Shanghai, China; 2 University of Chinese Academy of Sciences, Shanghai, China; 3 Beijing Institute of Biotechnology, Beijing, China; 4 Institute of Neuroscience, State Key Laboratory of Neuroscience, CAS Center for Excellence in Brain Science, Shanghai Institutes for Biological Sciences, Chinese Academy of Sciences, Shanghai, China; Charité—Universitätsmedizin Berlin, GERMANY

## Abstract

Mammalian circadian rhythm is established by the negative feedback loops consisting of a set of clock genes, which lead to the circadian expression of thousands of downstream genes *in vivo*. As genome-wide transcription is organized under the high-order chromosome structure, it is largely uncharted how circadian gene expression is influenced by chromosome architecture. We focus on the function of chromatin structure proteins cohesin as well as CTCF (CCCTC-binding factor) in circadian rhythm. Using circular chromosome conformation capture sequencing, we systematically examined the interacting loci of a Bmal1-bound super-enhancer upstream of a clock gene *Nr1d1* in mouse liver. These interactions are largely stable in the circadian cycle and cohesin binding sites are enriched in the interactome. Global analysis showed that cohesin-CTCF co-binding sites tend to insulate the phases of circadian oscillating genes while cohesin-non-CTCF sites are associated with high circadian rhythmicity of transcription. A model integrating the effects of cohesin and CTCF markedly improved the mechanistic understanding of circadian gene expression. Further experiments in cohesin knockout cells demonstrated that cohesin is required at least in part for driving the circadian gene expression by facilitating the enhancer-promoter looping. This study provided a novel insight into the relationship between circadian transcriptome and the high-order chromosome structure.

## Author Summary

Circadian rhythm regulates daily oscillations of many physiological processes in a wide range of organisms. In mammals, circadian rhythm drives the cycling expression of thousands of downstream genes. The temporal control of transcription takes place under high-order chromosome structure, which is established by looping distant loci on the linear DNA double strands. The most important chromatin structure proteins studied so far are cohesin and CTCF. Using circular chromosome conformation capture technologies, we found that cohesin binding sites are enriched in interacting regions of an enhancer bound by a key circadian transcription factor, Bmal1. Globally, cohesin and CTCF have disparate functions on transcriptional regulation. We developed a quantitative model integrating the effects of cohesin and CTCF in circadian gene regulation. With further computational and experimental approaches, we validated several cases of circadian oscillating genes where cohesin facilitates the enhancer-promoter looping. Taken together, this study showed that circadian gene expression is orchestrated under the long-range interactions mediated by cohesin.

## Introduction

Circadian rhythm is a daily oscillation of physiological processes and behaviors in varieties of living systems [1,2]. In mammals, the endogenous clock is established by interconnected transcriptional-translational feedback loops including a series of clock genes, for instance, *Bmal1*, *Clock*, *Nr1d1*, *Nr1d2*, *Per* and *Cry* family genes [3,4]. Transcription factor complex Bmal1-Clock drives *Nr1d1*, *Nr1d2*, *Per* and *Cry* family gene expression via cis-regulatory element E-box. Conversely, Per and Cry proteins repress the transcriptional activity of Bmal1-Clock by protein-protein interaction. In addition, transcription repressors Nr1d1 (Rev-erbα) and Nr1d2 (Rev-erbβ) inhibit the transcription of Bmal1 through retinoic acid-related orphan receptor response element (RRE). Other clock genes like *Dbp*, *Tef*, *Dec1*, and *Dec2* are also involved in the feedback loops. These genes constitute the molecular makeup of central clock system that robustly oscillates across different tissues and generate the circadian expression of thousands of downstream genes. In mammals, master clock residing in suprachiasmatic nucleus (SCN) directs tissue-specific circadian clocks in peripheral tissues. Circadian oscillating genes (COGs) showing 24-hour rhythm in mRNA expression level in mouse liver have been intensively studied by transcriptomic profiling technologies [5,6]. High-throughput studies on circadian transcription factor binding [6,7] and histone modifications [6,8] by ChIP-Seq, and enhancer RNAs by GRO-Seq [9] have hinted the circadian regulation in intergenic regions distal to gene promoters. Furthermore, the cycling profiles of many COGs were found to be inconsistent with the proximal binding of circadian transcription factors [10]. Thus, long-range chromasome interactions between promoters and enhancers may be required for a deeper understanding of the temporal organization of widespread COGs.

Over the past few years, the development of comprehensive chromosomal interaction mapping technologies facilitated our current understanding of three-dimensional architecture in chromosome conformation [11]. It was found that the boundaries of chromatin interaction domains are enriched for binding sites of CTCF (CCCTC-binding factor) [12,13], which is commonly accepted as a barrier protein binding to the insulators [14]. Cohesin is another chromosome structure protein with crucial function in sister chromatin cohesion and chromosome remodeling [15]. Cohesin complex contains four subunits, Smc1, Smc3, Scc1 (also called Rad21), and Scc3 (known as Stag1 and Stag2 in mammalian cells), which form an open-close ring structure to hold DNA [16,17]. Cohesin cooperates with Mediator or CTCF [18,19] in controlling gene expression independent of its function in sister chromatid cohesion [20]. The co-binding sites of CTCF and cohesin repress gene expression by insulating enhancer action [18,21]. In comparison, CTCF-independent cohesin binding sites are reported to be cell type specific and predominately associated with transcriptional factor binding sites [22,23].

The high-order chromosome structure conveys important message on the transcription [24], which should also apply to the regulation of COGs. An earlier study in mouse embryonic fibroblast (MEF) cells analyzed the chromosomal interactions anchored to a COG *Dbp* [25]. However, the roles of chromosome structure proteins were not yet explored. In this study, we systematically identified long-range interactions involving a Bmal1 bound super-enhancer upstream of a clock gene *Nr1d1* in mouse liver. Notably, we found that cohesin binding sites are enriched in these interactions. With bioinformatics analysis and further experiments in cohesin-deficient MEF cells, our study provides the first line of evidences that cohesin can exert the influence upon genome-wide circadian expression by mediating long-range chromosome interactions.

## Results

### The interactome of a circadian super-enhancer is enriched with cohesin

**Fig 1:**
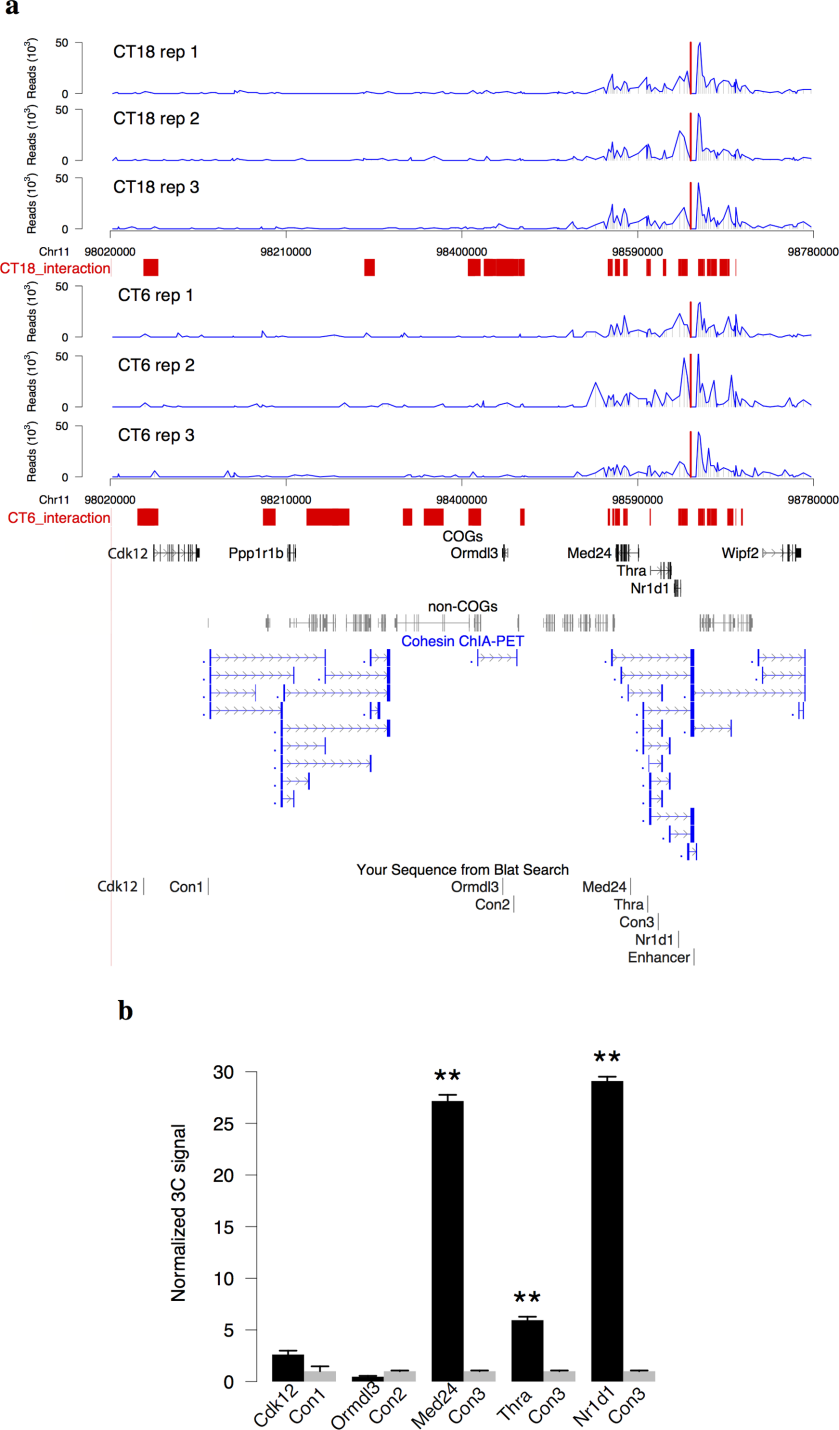
4C-Seq of a circadian super-enhancer. (a) The read profile of 4C-Seq at the enhancer locus upstream 8 kb of *Nr1d1* (red lines). Three mice were sacrificed for each time point (CT6 and CT18). The enhancer interacting regions were shown in red bars (Methods, FDR = 0.01 in at least two out of three replicates). COGs [5] and non-COGs in this region were showed respectively. The highly interacting region, 150 kb surrounding the bait, is enriched with cohesin loops from cohesin ChIA-PET in mouse ES cells [29]. (b) The result of 3C-qPCR assays between the bait and *Nr1d1*, *Thra*, *Med24*, *Ormdl3*, and *Cdk12* genes at CT6 (mean+/-SD, 3 biological replicates, 3 technical replicates). The positions of primers in 3C assays were shown at the bottom of Fig 1A. Student’s t-test was applied to compare 3C signals between gene promoter and control region. **p < 0.01.

To study the effect of high-order chromatin structure on circadian rhythm, we focus on a pioneer-like transcription factor in circadian regulation: Bmal1 [26]. We identified 3,244 Bmal1 enhancers [27] in mouse liver from published Bmal1 binding sites and histone marks of enhancers. Among them, the top 3% with highest Bmal1 binding signals were defined as super-enhancers [28] (Methods, S1 Table). To reveal the long-range interactions involved in circadian enhancers, we selected a Bmal1 super-enhancer located ~8 kb upstream of a clock gene *Nr1d1* (S1A Fig). This enhancer harbors the strongest Bmal1 binding site in mouse liver with rhythmic binding (S1B Fig). Using this enhancer as the bait, we detected its interacting regions in mouse liver by circular chromosome conformation capture sequencing (4C-Seq) at CT6 (CT: circadian time, n = 3) and CT18 (n = 3) when Bmal1 binding is at its peak and trough respectively. Genomic regions consistently enriched in 4C signals in at least two out of three biological replicates at a given time point were identified as enhancer interacting regions, resulting 49 regions at CT6 and 51 regions at CT18 respectively within 2 Mb to the enhancer (FDR = 0.01, Methods and S2 Table). A highly interacting region spanning approximately ~150 kb around the bait region shows markedly elevated signals at both CT6 and CT18 (Fig 1A and S1C Fig).

**Fig 2:**
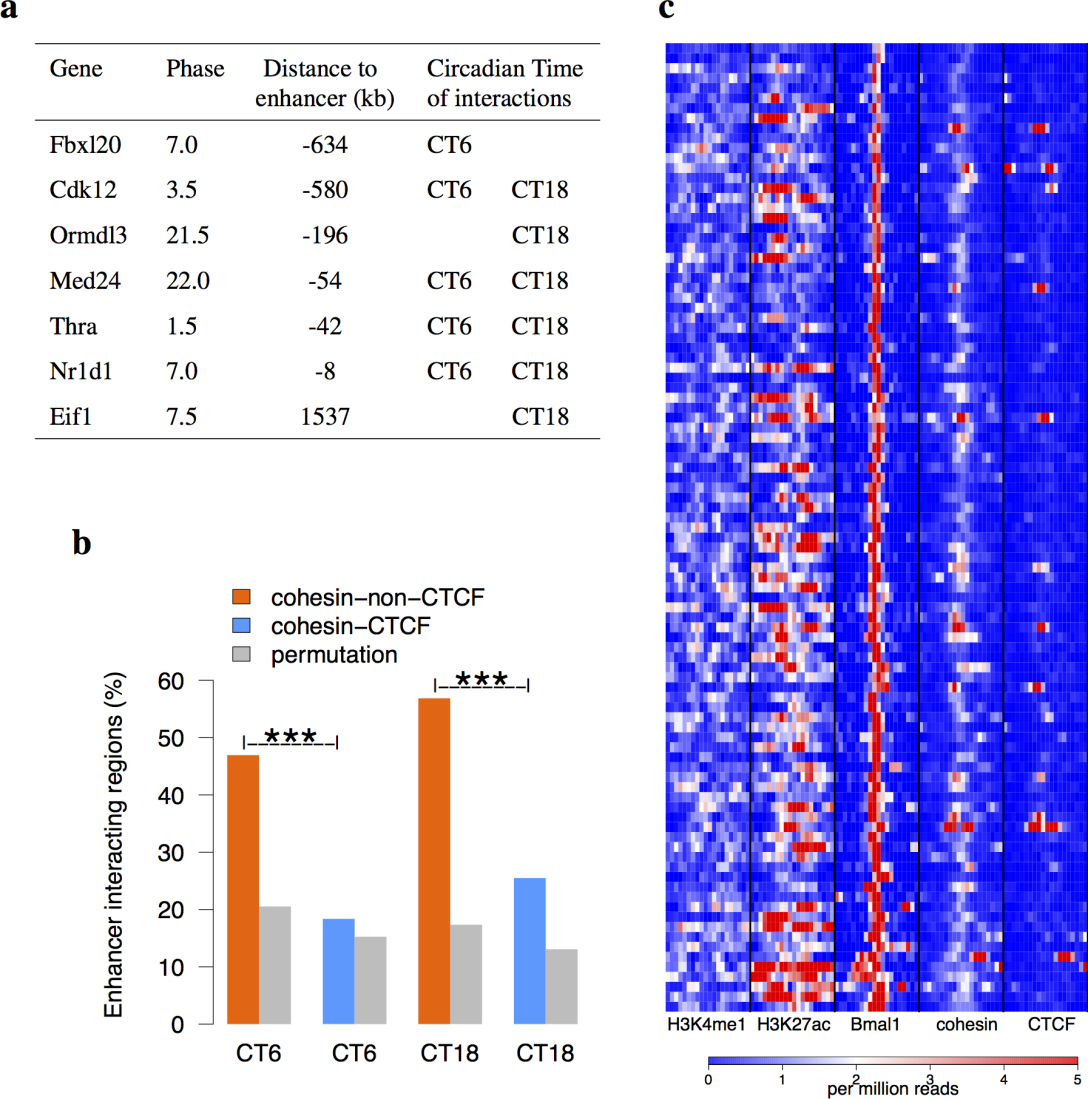
Cohesin is involved in circadian interactome. (a) The COGs in mouse liver interacting with the enhancer at CT6 or CT18. Their circadian phases and distances to the bait were listed. (b) The enhancer interacting regions significantly overlap more with the binding sites of cohesin-non-CTCF than cohesin-CTCF and randomly permutated cohesin-CTCF or cohesin-non-CTCF sites at both time points (binomial test). These two binding sites were identified from *de novo* analysis of ChIP-Seq data [22] (Methods). (c) The H3K4me1, H3K27ac [31], cohesin, and CTCF [22] ChIP-Seq signals around 97 Bmal1 super-enhancers. The binding sites were sorted by the signals on Bmal1 ChIP-Seq data [6]. The Bmal1 super-enhancers contain higher binding signals of cohesin than CTCF. ***p < 10^−4^.

We next obtained 3,018 COGs and their circadian phases from a published microarray data of high temporal resolution in mouse liver [5]. Out of them, *Fbxl20*, *Cdk12*, *Med24*, *Thra*, and *Nr1d1* show interactions with the enhancer at CT6. Quantitative chromosome conformation capture (3C-qPCR) analysis was performed to validate the interactions between selected COGs and the enhancer at CT6. In all cases tested, the interactions identified by 4C are highly consistent with 3C-qPCR results (Fig 1A and 1B). *Cdk12* shows a weak interaction with the enhancer. *Ormdl3*, a non-interacting COG at CT6, shows a lower interaction with the enhancer than the control. In comparison, *Nr1d1*, *Thra*, and *Med24* demonstrate strong interactions with the enhancer of 6–30 folds over the nearby control regions. The highly interacting region identified in our study falls into one of topologically associating domains (TAD) identified by Hi-C in mouse embryonic stem (ES) cells [12] (S2 Fig). The circadian phases of *Thra* and *Med24* are both around CT0 (Fig 2A). The closeness of their circadian phases suggests that they are likely co-regulated by the same enhancer [8]. Interestingly, the interactions with the bait within highly interacting region are significantly enriched of chromatin loops from cohesin ChIA-PET (chromatin interaction analysis by paired-end tag) data [29] (Fig 1A, Chi-squared test p < 10^−16^) but are devoid of chromatin loops from CTCF ChIA-PET data in mouse ES cells [30]. The interactome data in mouse ES cells implies the potential involvement of cohesin in the long-range interactions with *Nr1d1* enhancer. When we examined broader regions of interactions, nearly 50% of enhancer interacting regions at CT6 or CT18 overlapped with cohesin-non-CTCF sites as compared to merely 20% for cohesin-CTCF sites or random sites obtained by the permutation of cohesin-CTCF or cohesin-non-CTCF sites (Fig 2B). Furthermore, we profiled all Bmal1 super-enhancers in mouse liver and found that they have high occupancy of cohesin (Fig 2C). Therefore, our 4C-Seq data suggested that cohesin is implicated in facilitating circadian enhancer and promoter interactions.

### Cohesin-non-CTCF binding sites are associated with high circadian rhythmicity of transcription

To globally investigate the relationship between circadian gene expression and chromosome structure proteins, we collected circadian cistrome data consisting of different DNA-binding proteins including architectural proteins cohesin and CTCF [22], core circadian transcription factors Bmal1 and Nr1d1 [6], as well as a non-circadian transcription factor Gabpa [22] from published ChIP-Seq datasets in mouse liver (Methods). All datasets were analyzed from the raw data and with the same pipeline. None of the components of cohesin and CTCF are circadian oscillating in their expression levels in mouse liver [3]. Because of the distinct function of cohesin from CTCF [22], we further classified the cohesin binding sites into cohesin-CTCF co-binding sites and cohesin-non-CTCF binding sites. Compared to cohesin-non-CTCF, the number of CTCF-non-cohesin sites is much fewer and therefore has been omitted from the analysis. In total, we obtained 10,948, 28,883, 23,662, 41,690, and 32,899 binding sites for Bmal1, Nr1d1, Gabpa, cohesin-CTCF, and cohesin-non-CTCF in mouse liver respectively.

**Fig 3:**
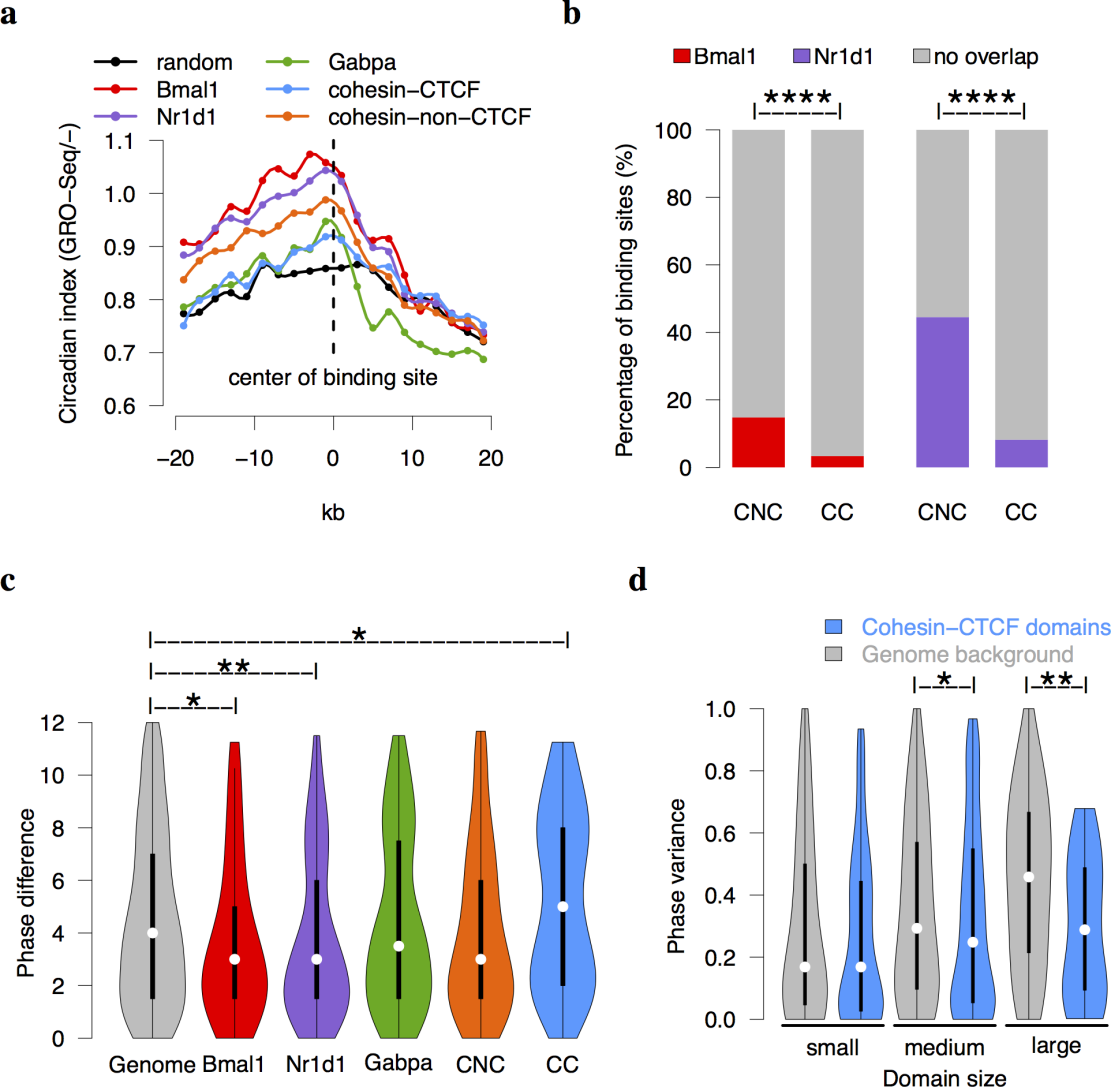
Global analysis of cohesin and CTCF on circadian transcription. (a) The distribution of circadian rhythmicity of transcription around the protein binding meta-sites (Methods). The circadian index was defined from the negative strand of GRO-Seq [9]. The result from positive strand was shown in S3A Fig. The data points are connected by spline smoothing method. The circadian rhythmicity is high around Bmal1, Nr1d1, and cohesin-non-CTCF sites but not around cohesin-CTCF sites. (b) Both Bmal1 and Nr1d1 binding sites have significantly higher overlaps with cohesin-non-CTCF sites (CNC) than cohesin-CTCF (CC) sites in mouse liver (Fisher’s exact test p = 10^−16^). (c) The distributions of phase differences of neighboring COGs are shown in a violin plot. The phase differences across Bmal1 and Nr1d1 binding sites are significantly smaller than the genomic background, whereas the phase differences across cohesin-CTCF sites are larger than the genomic background (Mann-Whitney U test). (d) The violin plot of phase variance in cohesin-CTCF domains. The medium and large cohesin-CTCF domains inferred from cohesin ChIA-PET data [29] have smaller phase variances than the background domains (Mann-Whitney U test). ****p < 10^−8^,**p < 0.01, *p < 0.05.

We defined a nucleotide-level circadian index using circadian time-series GRO-Seq data [9] to quantify circadian transcriptional activities across whole mouse genome in mouse liver (Methods). In our definition, higher circadian index indicates stronger rhythmicity. As expected, the binding centers of Bmal1 and Nr1d1 have overall higher circadian indices than the other factors or genomic background. Interestingly, the profile of cohesin-non-CTCF sites is between Bmal1/Nr1d1 and the random sites, which implicates a positive role of cohesin-non-CTCF on circadian rhythmicity (Fig 3A, S3A Fig). This phenomenon is again observed when defining circadian index using circadian time-series RNA-Seq data [6] (S3B Fig). Moreover, both Bmal1 and Nr1d1 binding sites prefer to overlap with cohesin-non-CTCF binding sites rather than cohesin-CTCF binding sites in mouse liver (Fisher’s exact test p < 10^−22^, Fig 3B). Therefore, cohesin-non-CTCF sites are associated with high circadian rhythmicity of transcription.

### Cohesin-CTCF co-binding sites insulate circadian phases of COGs

Cohesin-CTCF co-binding sites are known to play the role of genomic insulator [32]. To study whether cohesin-CTCF sites affect the circadian gene expression in mouse liver, we compared the phase differences of two neighboring COGs separated by a given binding site to the genomic background (Methods, S3C Fig). The phase differences of two adjacent windows were significantly smaller than the phase differences of two windows that were randomly picked from the genome (Mann-Whitney U test p = 10^−10^, S3C Fig). This demonstrated that the neighboring COGs across the genome tend to have similar circadian phases. Interestingly, the phase differences across cohesin-CTCF sites show a bimodal distribution and are significantly larger than those in genomic background (Mann-Whitney U test p = 0.03, Fig 3C). On the contrary, Bmal1 and Nr1d1 binding reduced the phase differences of COGs across their binding sites (Mann-Whitney U test p = 0.03 and 0.006 respectively). It indicated that Bmal1 and Nr1d1 might lead to the oscillation of genes in the similar phases in both directions flanking the binding sites. The effect of cohesin-non-CTCF was again similar to those of Bmal1 or Nr1d1, although it is only moderately statistically significant from the background (Mann-Whitney U test p = 0.1). In comparison, the distribution of phase differences across Gabpa sites was similar to that of genomic background. These results revealed that cohesin-CTCF co-binding sites tend to disrupt the phase continuity of neighboring COGs.

We then asked whether the COGs within the same domain defined by interacting cohesin-CTCF sites show similar circadian phases. Due to the lack of cohesin-CTCF domains in mouse liver, we inferred tissue/cell type invariant cohesin loops from the ChIA-PET data in mouse ES cells [29] in which only loops with both anchors overlapped with cohesin-CTCF binding sites in mouse liver were selected (S3D Fig). The variance of circadian phases was used to measure the phase difference of two or more COGs. We observed that the phase variance of genomic background increases as an exponential function of the domain size (S3D Fig, p < 10^−16^, Pearson’s correlation coefficient = 0.37). To take into account of this size effect, we divided cohesin-CTCF domains into three categories according to their sizes and compared to genomic background in the corresponding sizes (Methods). The phase variances were smaller in cohesin-CTCF domains of medium and large sizes compared to genomic background at significance levels of p = 0.01 and 0.003 respectively (Fig 3D, Mann-Whitney U test). In summary, the chromosomal domains defined by cohesin-CTCF co-binding sites tend to lock the phases of COGs.

### A cohesin/CTCF dependent model of circadian gene regulation

**Fig 4:**
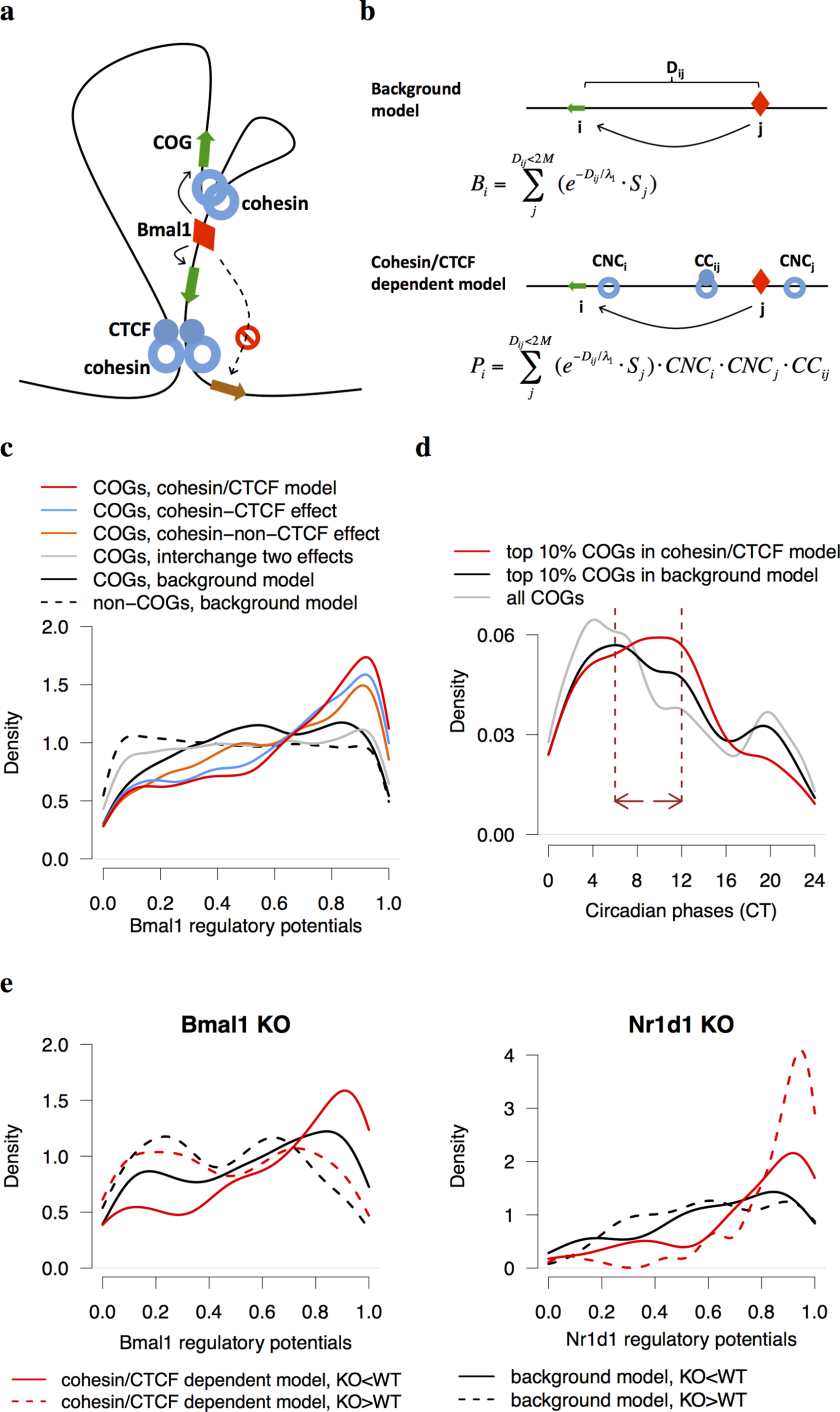
A model of circadian regulation of gene expression under high-order chromosome organization. (a) Cohesin-CTCF disrupts the gene regulation of circadian transcription factors, whereas cohesin-non-CTCF facilitates the contacts between circadian enhancers and promoters. (b) In the background model, only Bmal1 binding sites within 2 Mb of the genes were considered. In the cohesin/CTCF dependent model, the contribution of each Bmal1 binding site was further multiplied by three cohesion/CTCF dependent factors: *CNC*_*i*_, *CNC*_*j*_, and *CC*_*ij*_ (Methods). (c) The density plot of regulatory potentials in COGs or non-COGs under different conditions. The COGs in cohesin/CTCF dependent model, as well as in the model with only cohesin-CTCF or cohesin-non-CTCF effect, have significantly higher Bmal1 regulatory potentials than the background model (Kolmogorov-Smirnov test p = 10^−16^). The regulatory potential of the model where cohesin-non-CTCF sites and cohesin-CTCF sites were interchanged is similar with the background model. (d) The density plot of phases of COGs top ranked in two models. The enriched phase regime (CT6-CT12, dash lines) of COGs with top 10% Bmal1 regulatory potentials in cohesin/CTCF dependent model follows the peak of Bmal1 binding at CT6. (e) The density plots of differentially expressed genes in *Bmal1* and *Nr1d1* KO datasets. Under-expressed genes in *Bmal1* KO (Fan et al., manuscript in preparation) have higher Bmal1 regulatory potentials in cohesin/CTCF dependent model than those in the background model, whereas over-expressed genes in *Nr1d1* KO [9] have markedly increased in Nr1d1 regulatory potentials.

In light of the above observations, we proposed a model that incorporated the effects of cohesin mediated enhancer-promoter interactions on the gene regulation in chromosomal domains defined by the co-binding of cohesin and CTCF (Fig 4A). We adopted the concept of regulatory potential to quantify the regulation of a gene by a given circadian transcription factor [33]. The regulatory potentials of Bmal1 on all annotated genes in mouse genome were calculated with or without considering the effect of cohesin and CTCF (Methods, Fig 4B and S3 Table). In the background model, the regulatory potential *B*_*i*_ of Bmal1 on a given gene *i* was computed as the sum of contributions from all available Bmal1 binding sites *j* within 2 Mb of the gene, that is, $B_{i}=\sum_{D_{\text{ij}}<2Mb}\left(e^{-D_{ij}/\lambda_{1}}\cdot S_{j}\right)$, where *D*_*ij*_ is the distance between gene *i* and Bmal1 binding site *j* and *S*_*j*_ is the strength of Bmal1 binding at site *j*. Here we assumed that the regulatory effect of transcriptional factor on its target gene decays exponentially with distance from the binding site to its target gene and *λ*_*1*_ is the characteristic distance. In the cohesin/CTCF dependent model, the contribution of gene *i* and Bmal1 binding site *j* was further multiplied by three factors corresponding to the enhancing effects of a cohesin-non-CTCF site either near gene *i* (*CNC*_*i*_) or a Bmal1 binding site *j* (*CNC*_*j*_) as well as the insulating effect of cohesin-CTCF site (*CC*_*ij*_), i.e. $P_{i}=\sum_{D_{\text{ij}}<2Mb}(e^{-D_{ij}/\lambda_{1}}\cdot S_{j})\cdot {CNC}_{i}\cdot {CNC}_{j}\cdot {CC}_{ij}$, (Methods). At last, the regulatory potentials were normalized to the ranks across all genes to ensure the robustness of model parameters.

Comparing with the background model, the regulatory potentials of Bmal1 on COGs were significantly higher in the cohesin/CTCF dependent model (Fig 4C, Kolmogorov-Smirnov test p = 10^−16^). It was known that the circadian phase of Bmal1 binding occurs around CT6 [7] and the phases of COGs directly controlled by Bmal1 are typically between CT6 and CT12. We found that the phases of COGs with top ranked Bmal1 regulatory potentials in cohesin/CTCF dependent model are more enriched in CT6-CT12 following the binding peak of Bmal1 at CT6 compared to the background model (Fig 4D). Using Nr1d1 ChIP-Seq data, we observed that Nr1d1 regulatory potentials in cohesin/CTCF dependent model could also distinguish COGs from non-COGs (S4A and S4B Fig). The fact that most core circadian clock genes have higher regulatory potentials in cohesin/CTCF dependent model suggested that chromosome structure proteins might facilitate the transcription of core components of circadian clock (S4C Fig). Taken together, our cohesin/CTCF dependent model is a more sophisticated model that integrated circadian transcription factors and chromatin organizers to explain the circadian gene expression.

To validate the regulatory potentials of circadian transcription factors, we examined the differentially expressed genes in the livers of *Bmal1* knockout (KO) (Fan *et al*., manuscript in preparation) and *Nr1d1* KO mice [9]. We observed that under-expressed genes in *Bmal1* KO have higher Bmal1 regulatory potentials in cohesin/CTCF dependent model than those in the background model, while over-expressed genes in *Bmal1* KO have similar regulatory potentials between two models (Fig 4E). In contrast, over-expressed genes rather than under-expressed genes in *Nr1d1* KO showed much higher Nr1d1 regulatory potentials in cohesin/CTCF dependent model than those in the background model (Fig 4E). This is consistent with the current notion that Bmal1 functions as an activator and Nr1d1 as a repressor in circadian regulation.

### Gene expression changes upon *in vitro* cohesin knock-out

**Fig 5:**
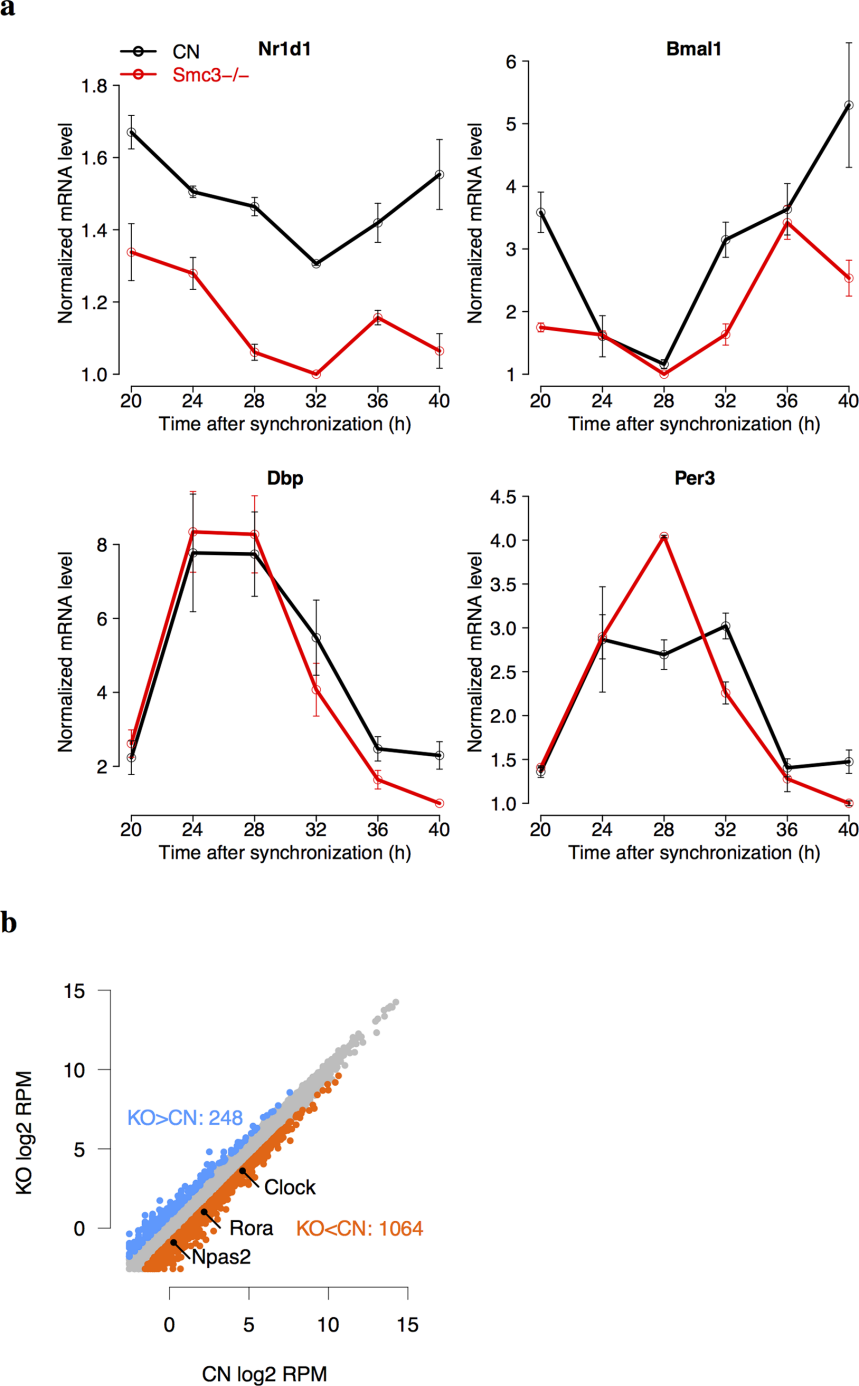
Cohesin knockout in MEFs. (a) The RT-PCR results of the expression of clock genes in control (CN) and *Smc3-/-* (KO) MEFs. Gene expression were measured every 4 hr for 24 hr after the cells were synchronized by 1-hr dexamethasone treatment at CT0. Each data point contained two biological replicates and three technical replicates. All four clock genes showed significant circadian expression in CN and KO by cosine fitting (p < 0.05) except for *Nr1d1* in KO. ANOVA analysis showed that the expression of *Nr1d1* has significant difference between CN and KO (p = 10^−7^). (b) The scatterplot of global gene expression from RNA-Seq in *Smc3*-/- and control MEFs. 20-hr and 32-hr samples were combined together to determine the differentially expressed genes. Core clock genes including *Clock*, *Rora*, and *Npas2* were under-expressed in cohesin KO.

The knock-out of cohesin subunits, Smc3, Scc1, and Scc3, lead to the embryonic lethality in mice [34]. To establish a knock-out system of cohesin *in vitro*, we transfected the post-mitotic *Smc3*-flox/flox MEF cells by Cre/GFP adenovirus such that the expression of *Smc3* decreased by 80–90% in *Smc3*-/- cells compared to control cells (Methods). We measured the mRNA levels of four clock genes in *Smc3*-/- cells by RT-PCR assays after synchronizing the cells with dexamethasone treatment (Fig 5A). All genes showed significant oscillations both in KO and control cells (cosine fitting, p < 0.05) except for *Nr1d1* in KO cells. *Nr1d1* showed under-expression in KO cells (ANOVA, p = 10^−7^). The peak-trough ratio of *Bmal1* dropped from 3.7 in control to 2.4 in KO cells. The circadian oscillations of *Dbp* and *Per3* were not affected upon cohesin KO. Although the core clock genes have consistent cycling expression *in vivo* across tissues [3], the number of circadian oscillating genes *in vitro* in cell lines is much fewer than *in vivo*. To examine the gene regulation of circadian transcriptional factors in MEFs, we conducted Bmal1 ChIP-seq data in control MEF cells (Methods). However, only 244 Bmal1 binding sites were identified (S4 Table) including those on the promoters of core clock genes, *Nr1d1* (S1A Fig), as well as *Nr1d2*, *Cry1*, *Cry2*, *Per1*, *Bhlhe41*, and *Dbp* (S4 Table). The lack of Bmal1 binding sites on most hepatic COGs is consistent with the fact that they are not oscillating in synchronized MEF cells.

To reveal the broader impact of cohesin on gene expression, we then applied RNA-Seq to measure gene expression in *Smc3*-/- MEFs vs. control MEF cells. In total, 248 and 1,064 genes were identified as over-expressed and under-expressed genes respectively in cohesin KO (log2 fold change > 0.8, Fig 5B and S4 Table). The promoter regions of differentially expressed genes upon cohesin KO were enriched with cohesin binding sites in MEFs (Fisher’s exact test p = 0.002). Interestingly, the genes involved in circadian clock were significantly enriched among the under-expressed genes by Gene Set Enrichment Analysis [35] among the canonical pathways (FDR = 10^−8^). To extrapolate our result in cohesin KO MEFs to mouse liver, we next focus on tissue/cell type invariant enhancer-promoter interactions mediated by cohesin. We found that 22% of differentially expressed genes in cohesin KO have their promoter regions situated near an anchor of cohesin loops in mouse ES cells [29], suggesting they are regulated by invariant enhancer-promoter loops. To identify the invariant cohesin loops, we required that both anchors of the cohesin loop in ES cells are also bound by cohesin in mouse liver. Furthermore, one anchor of the loop is situated within 15 kb near either a Bmal1 or Nr1d1 binding site in liver and the other anchor resides within 5 kb near the transcription start site of a hepatic COG that was also differentially expressed in cohesin KO in MEFs. We also required that the circadian phases of candidate genes fall into either Bmal1 controlled phase regime (CT6-CT12) or Nr1d1 controlled phase regime (CT20-CT2). The candidate pairs of COGs and enhancers identified were listed in Table 1.

**Table 1:** The candidate COGs interacting with circadian enhancers via invariant cohesin-mediated loops.

Gene Symbol	Circadian Phase (CT)	Transcription Factor	Transcription Factor binding site position (kb)	Cohesin KO-CN Log2-Fold-Change (LFC)
*Tmtc2*	11	Bmal1	200	-2.5
*Rnf43*	7.5	Bmal1	39	-1.5
*Phldb2*	7	Bmal1	126	-1.1
*Cdo1*	6	Bmal1	-130, -121	-1.2
*Kcnb1*	11	Bmal1	-128	-1
*Atr*	11	Bmal1	-317,-315,-305	-1.4
*1200009I06Rik*	23.5	Nr1d1	-30	-0.8
*Ahnak*	20.5	Nr1d1	106, 108, 131	-0.9
*Dapk1*	23.5	Nr1d1	-121, -116, 129, 135	-1.2
*Npas2*	1	Nr1d1	-179, -169	-1.1

**Fig 6:**
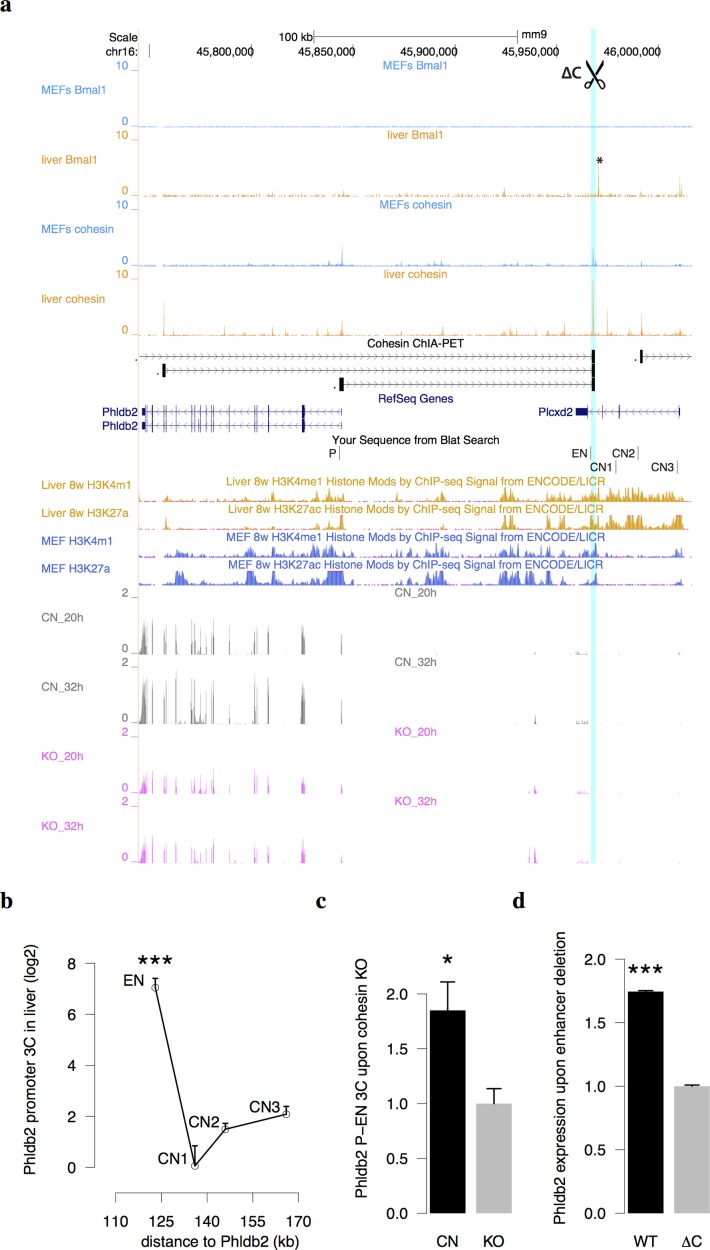
The expression of a COG, *Phldb2*, is influenced by an invariant cohesion-mediated enhancer-promoter interaction. (a) A cohesin loop connects the BMAL1 enhancer (marked by asterisk) and the promoter of *Phldb2*. Conserved cohesin binding sites and active histone marks between liver and MEFs were found at the promoter of *Phldb2* and its enhancer. The enhancer was bound by Bmal1 in liver but not in MEFs. The locations of primers used in 3C assay in liver and MEFs were indicated below the RefGenes. ∆C denotes CRISPR-mediated deletion region. The normalized RNA-Seq in *Smc3*-/- and control MEFs in this region were showed in the bottom. (b) The 3C signals of interactions anchored to *Phldb2* promoter in mouse liver (ANOVA p = 10^−7^, mean+/-SD, 2 biological replicates, 4 technical replicates). CN, control. EN, enhancer. (c) The 3C signals of *Phldb2* promoter-enhancer interaction in control and *Smc3*-/- MEFs (t-test p = 0.04, mean+/-SD, 2 biological replicates, 3 technical replicates). (d) The normalized expression level of *Phldb2* after the deletion of cohesin binding site near the enhancer of *Phldb2* in Hepa1-6 cells (t-test p = 10^−11^, mean+/-SD, 2 biological replicates, 3 technical replicates). ***p < 10^−4^, *p < 0.05.

We then used 3C-qPCR experiments to confirm the presence of enhancer-promoter interactions in two such cases, *Phldb2* and *Ahnak* in mouse liver (Fig 6B and S5B Fig). We also found that both interactions were significantly weakened in cohesin KO MEF cells compared to control cells (Fig 6C and S5C Fig). *Phldb2* encodes a microtubule-anchoring factor that binds to phosphoinositides and filamin [36]. *Phldb2* shows circadian phase at CT7 in mouse liver and is interacting with a Bmal1-bound enhancer situated 126 kb upstream in the intron of another gene *Plxd2* (Fig 6A). This Bmal1 binding site is confirmed by ChIP-PCR in mouse liver (S6A Fig). Ahnak protein is a mediator in calcium signaling and transforming growth factor β signaling pathways [37]. *Ahnak* shows circadian phase at CT21 and is interacting with an Nr1d1-controlled enhancer (S5A Fig). The promoters of *Phldb2* and *Ahnak* are devoid of any Bmal1 or Nr1d1 binding sites in liver. Furthermore, we found conserved histone modification marks of active transcription and cohesin binding sites at these two genes and their enhancer loci in both MEFs and liver. This supports that the cohesin-mediated loops in *Phldb2* and *Ahnak* are invariant between tissues or cell types. Finally, to show that these interactions are functional for gene regulation, we used CRISPR-CAS9 system to delete the cohesin binding site near the enhancer of *Phldb2* in Hepa1-6 cells and found a significant reduction of 41% in the expression of *Phldb2* (Fig 6D and S6B Fig). *Phldb2* is not circadian oscillating in synchronized MEFs or Hepa1-6 cells due to the lack of Bmal1 binding in their enhancers (Fig 6A). The binding of Bmal1 on the enhancer of *Phldb2* renders its circadian expression in liver. Taken together, our results suggest that the stable and invariant enhancer-promoter loop mediated by cohesin is a prerequisite for temporal gene regulation in circadian rhythm.

## Discussion

In complex organisms, it is known that genome-wide transcription is highly organized under high-order chromosome structure. In particular, distal enhancer has been considered to play a key role in gene regulation through long-range interactions. Given its far-reaching effect on gene expression, circadian clock is an ideal system to investigate the interplay between chromosome architecture and temporal regulation of gene expression under homeostasis. It was proposed that orchestrated transcription takes place at the so-called “transcription factories” where genes from distant loci across the genome are physically in contact. COGs within the same transcription factory may be regulated under the common circadian regulators such as Bmal1. Our 4C-Seq data for a super-enhancer upstream of *Nr1d1* provided evidence of physical interactions between the enhancer and multiple COGs. This super-enhancer contains the binding sites of both Bmal1 and Nr1d1 (S1A Fig), a common feature in circadian cistrome [6]. Among the interacting genes within 2 Mb of the super-enhancer, the circadian phases of *Fbxl20*, *Nr1d1*, and *Eif1* follows the phase of Bmal1 binding, while the phases of *Ormdl3*, *Med22*, and *Thra* suggests that they are more likely co-regulated by Nr1d1 (Fig 2A). We also found that strong interactions within 150 kb of the super-enhancer were independent of circadian time and restricted in a cell type invariant TAD. These results hinted that the long-range interaction acts as a stable backbone rather than a dynamic driving force for circadian regulation. Similar findings of stable interactions have been reported in other temporal processes such as animal development [38] although chromosome domains are highly dynamic during the stages of cell cycle [39]. Comparing with cohesin ChIA-PET data in mouse ES cells [29], we found the presence of multiple cohesin-mediated loops coinciding with the highly interacting regions of the enhancer. The enrichment of cohesin binding signals in both 4C interacting regions and on Bmal1 super-enhancers conforms to the general role of cohesin in organizing genome structure for gene regulation, although this may not be unique for circadian regulation.

The availability of cohesin and CTCF ChIP-Seq data in mouse liver provided us a unique opportunity to investigate the genome-wide association between cohesin and CTCF binding sites with circadian genomic features. To examine the effect of cohesin in circadian system, we designed a pipeline to capture the continuous change of circadian rhythmicity of transcription across the binding sites of several proteins including Bmal1, Nr1d1, Gabpa, CTCF, and cohesin at 2 kb resolution. This unbiased approach allowed us to include un-annotated transcripts as well as unconventional transcription that does not take place from transcription start sites [40]. We observed that the profile of cohesin-non-CTCF binding sites resembles that of Bmal1 as compared to other non-circadian transcription factors. It suggests that cohesin-non-CTCF sites have a positive effect on circadian rhythmicity of transcription although cohesin itself is not known to be a circadian regulator. From circadian phase analysis, we noted that neighboring circadian genes tend to have similar circadian phases while the co-binding of CTCF and cohesin leads to the insulation of circadian phases. Our finding is consistent with a recent study reporting that CTCF attenuates the transcription of circadian oscillating genes by mediating their contacts to the nuclear lamina [41]. Previous study showed that the nearest genes around Bmal1 sites were not always rhythmically expressed or peaking in the phase regime predicted for a Bmal1 controlled gene [7]. Based on 4C-Seq and bioinformatics analysis, our model incorporating the disparate effects of cohesin-non-CTCF and cohesin-CTCF provides a better predictor of the circadian expression of genes and their phases.

In this study, we have utilized a range of datasets from mouse liver and several cell lines. We chose mouse liver for our main analysis because it shows genome-wide circadian oscillation of gene expression [5] and it has the most comprehensive circadian transcriptomic and cistromic data to date. Mouse cell lines were used when genetic manipulations are not possible in liver as cohesin-deficiency is embryonic lethal *in vivo* [34]. Although synchronized MEF cells have been widely used for circadian studies [25,42–44], there are much fewer genes oscillating in MEFs and only core circadian genes are oscillating in both liver and MEFs. This is largely due to the lack of Bmal1 binding sites in MEFs as shown by our Bmal1 ChIP-Seq data in MEFs. For this reason, we used cohesin-deficient MEFs only to select candidate genes regulated by the invariant enhancer-promoter interactions mediated by cohesin even if these genes including *Phldb2* and *Ahnak* are not oscillating in MEFs. However, the histone marks and cohesin binding sites were very conserved on the enhancer loci of the two cases between liver and MEFs indicating these are tissue/cell type invariant TADs. This is further supported by the cohesin ChIA-PET data in ES cells even though ES cells lack a functional circadian clock [45]. We used Hepa1-6 cells here because of the convenience for CRISPR-CAS9 experiment in these cells. These data in cell lines collectively suggest that these invariant enhancer-promoter interactions are both cohesin-dependent and functional in gene regulation. These DNA loops were confirmed to be also present in liver and the binding of Bmal1 in these enhancers renders the circadian expression to these two genes in liver. This picture is in line with our model that cohesin-mediated enhancer-promoter loop provides a stable and tissue/cell type invariant backbone and circadian gene regulation is a result of dynamic Bmal1 binding on the stable chromosome structure. We are also aware that the DNA loops mediated by architectural proteins seem to be developmentally regulated at specific loci within the TADs [46]. Whether our finding has general applicability for long-range circadian regulation still awaits future studies with other experimental strategies. Overall, our study sheds new light on the transcriptional landscape of circadian genes under high-order chromosome structure.

## Methods

### Ethics statement

All animal experiments performed in this study were approved by the Institutional Animal Care and Use Committee of Shanghai Institutes for Biological Sciences and conformed to institutional guidelines of vertebrates study.

### Identification of Bmal1 bound super-enhancers

The general strategy for screening Bmal1 bound super-enhancers followed the pipeline described in [28]. We first defined 3,244 Bmal1 enhancers in mouse liver with the following rules: (1) the co-occurrence of H3K4me1 and H3K27ac marks [47,48], (2) positioning at least 1 kb away from any transcription start sites of annotated genes [49], (3) overlapping with Bmal1 binding sites at ZT8 from Koike et al.’s data [6] (see Methods section ChIP-Seq data analysis), (4) at least 100 bp in length. H3K4me1 and H3K27ac ChIP-Seq data in the livers of eight-week-old mice were used [31]. Because the signals on Bmal1 binding site do not show broad distribution, we skipped the step of merging enhancers in close distance. To obtain confident super-enhancers, the read numbers per million reads per kilobase from Koike et al.’s and Rey et al.’s Bmal1 ChIP-Seq experiments were added to rank Bmal1 enhancers [6,7]. Finally, 97 Bmal1 enhancers ranked at top 3% were defined as Bmal1 super-enhancers in mouse liver.

### Circular chromosome conformation capture sequencing (4C-Seq)

4C-Seq assays were performed as previously described [50,51] with modifications. Briefly, six-week-old male C57BL/6 mice were entrained to 24 hr cycles of 12 hr light and 12 hr dark for one week and then switched into constant darkness. Three mice each were sacrificed in the dark at CT6 and CT18, respectively. Mouse liver cells were quickly dispersed and filtered through the 40 mm cell strainer to make a single-cell suspension. Approximately 50-million cells were fixed in 1% formaldehyde for 10 min at room temperature before being quenched with 0.125 M glycine. Cells were then lysed in cold lysis buffer (10 mM Tris HCl, 10 mM NaCl, 0.2% NP-40, 1×protease inhibitor) for 15 min on ice. After being washed twice, cell nuclei were re-suspended in Buffer 2.1 (New England Biolabs) including 0.1% SDS and were incubated for 10 min at 65°C. 1% (final concentration) of Triton X-100 was added to quench SDS and centrifuged to remove SDS and Triton. Nuclei were then digested overnight by 800U HindIII (New England Biolabs) at 37°C with shaking. After inactivation by 1.6% (final concentration) of SDS at 65°C for 20 min, samples were washed and re-suspended in ligation buffer and ligated by 100U T4 DNA ligase (Thermo Fisher Scientific) at 16°C for 4 hr and then room temperature for 30 min. Ligated chromatin was digested by proteinase K before DNA purification. The purified DNA was further digested by DpnII (New England Biolabs) and then circularized using T4 DNA ligase (Thermo Fisher Scientific). After purification, 200 ng of DNA from the 4C library was used as the template for the PCR amplification using DyNAzyme EXT (Finnzymes). Primers specific to bait region (S5 Table) were applied to amplify the interactome of interest in a 25 μl reaction volume under the following PCR conditions: 1 cycle at 94°C for 2 min; (94°C 30 sec; 60°C 30 min; 72°C 2 min) ×18 cycles; 1 cycle of 72°C 7 min. PCR products (1 μl) were used as the templates for a second PCR reaction utilizing the primers with the addition of Illumina adaptors in a 50 μl volume under the same PCR conditions. The PCR-amplified library was purified and sequenced with a 100 bp read length using Illumina HiSeq 2000 (S6 Table).

Sequencing reads of 4C-Seq were de-multiplexed using the bait primers, *i*.*e*. removing the upstream of HindIII restriction site (AAGCTT) and the downstream of DpnII restriction site (GATC). Then the reads were aligned to mouse genome (mm9) by Bowtie [52]. The self-ligated reads and non-cut reads were removed [53]. Only the reads uniquely mapped to the HindIII restriction sites on the cis-chromosome of the bait were kept and assigned the HindIII restricted fragments defined by two neighboring restriction sites. Peak calling was performed with a custom-designed pipeline generally following FourSig [54]. Previous interactome studies reported that 99% interactions were less than 1 Mb and inter-chromosomal interactions were hard to be validated [55]. Hence, we only considered intra-interactions within 2 Mb of the bait. The highly interacting region (150 kb to the bait, S1C Fig) was masked out during the peak calling on other regions. A sliding window with size of 3 fragments was used to calculate the cutoff based on the comparison between 100 permutations of raw reads and true data. The distribution of cutoffs under FDR = 0.01 was profiled and the final cutoff was determined as the 95% quantile. For highly interacting region, this cutoff was multiplied by the reads ratio between highly interacting region and other regions. Then the merged peaks in highly interacting region and other regions were considered as the peaks in each sample. We required that the peaks at each time point were consistently called in at least two out of three biological duplicates. In total, 49 and 51 peaks were obtained at CT6 and CT18 respectively. To compare highly interacting regions with ChIA-PET, we selected 1000 random regions of the same size and applied Chi-squared test to evaluate the significance between overlapped loops in highly interacting regions and in the random regions. The Gene Expression Omnibus (GEO) accession number for the 4C dataset is GSE68830.

### Quantitative chromosome conformation capture (3C-qPCR)

3C-qPCR was performed as previously described with modifications [56]. Briefly, 10 μg of cross-linked nuclei were collected and shaken in 1 ml lysis buffer (1% SDS, 0.5% TritionX-100, proteinase inhibitor cocktail in TE buffer) for 1 hr at 37°C, followed by centrifugation for 3 min at 1000 rpm at room temperature. After removing the supernatant, the pellet was re-suspended in 500 μl digestion buffer (1% TritonX-100, 1xRE buffer, PI, 20 μl Quickcut HindIII in H_2_O) and digested overnight at 37°C with shaking. The reaction was terminated by the addition of SDS at a final concentration of 1.5% and the incubation at 65°C for 30 min. SDS and RE buffer were removed by centrifugation and the pellet was re-suspended for the next ligation. Reverse crosslinking was performed in the presence of proteinase K at 60°C overnight followed by RNaseA treatment at 37°C for 1 hr. The genomic DNA was extracted by phenol-chloroform. All 3C primers were designed by Primer Premier 6 (S5 Table).

### ChIP-Seq data analysis

The ChIP-Seq data of CTCF, Rad21, Stag1, Stag2, and Gabpa in mouse liver were downloaded from ArrayExpress database (accession: E-MTAB-941) [22]. The ChIP-Seq data of Bmal1 at CT8 in mouse liver was downloaded from Gene Expression Ominbus (GEO) database (accession: GSE39860) [6]. The ChIP-Seq data of Nr1d1 at CT10 in mouse liver was downloaded from GEO (accession: GSE26345) [57]. The ChIP-Seq data of CTCF and Smc1 in MEFs were downloaded from GEO (accession: GSE22557) [19]. Rad21, Stag1, Stag2, and Smc1 are the subunits of cohesin. Gabpa is a non-oscillating transcription factor in mouse liver chosen as a negative control. It is known that Bmal1 and Nr1d1 rhythmically bind to the genome and their binding peaks are around CT6 and CT10, respectively.

To ensure that different datasets are directly comparable, all these ChIP-Seq data were analyzed in the same pipeline described as below. Raw reads in FASTQ files were mapped on mouse genome (mm9 assembly) by Bowtie [52]. Only reads uniquely mapped with no more than two mismatches were considered as valid reads. Peak calling was implemented by MACS with default parameters and cutoff p < 10^−5^ [58]. The signal files generated from MACS were normalized to per million total reads. Broad peaks with multiple peaks were split to accurately determine the peak region by PeakSplitter [59], requiring per million reads larger than 1. Peaks generated from PeakSplitter were considered as the binding sites and the centers of peaks were considered as the binding centers. The binding sites of Smc1 were considered to represent the binding sites of cohesin in MEFs. The binding sites of cohesin in liver were defined as the union of binding regions of Rad21, Stag1, and Stag2. Consequently, we obtained 10,948, 28,883, 23,662, 50,683, and 74,589 binding sites for Bmal1, Nr1d1, Gabpa, CTCF, and cohesin in mouse liver respectively. In MEFs, we obtained 5,738 and 8,756 binding sites for CTCF and cohesin respectively. These cohesin binding sites that overlap with CTCF binding sites in liver were defined as cohesin-CTCF sites (41,690) and the cohesin binding sites not overlapping with CTCF binding sites were defined as cohesin-non-CTCF sites (32,899).

### Circadian rhythmicity of transcription

GRO-Seq (accession: GSE59486) [9] and RNA-Seq (accession: GSE39860) [6] data in mouse liver sampled every 3 or 4 hours over 1 day or 2 days were downloaded from GEO to obtain the genome-wide circadian gene expression. For each DNA binding factor including Bmal1, Gabpa, CTCF, and cohesin, the upstream 20 kb and downstream 20 kb relative to the binding centers were extracted. These regions were further divided into 2 kb bins as the basic unit for analyzing circadian rhythmicity of transcription across the genome. The 2-kb bin was considered as a valid bin if it contains at least one read at more than 7 (GRO-Seq) or 10 (RNA-Seq) time points. To exclude the binding sites in the region without any transcript, the binding site was considered for downstream analysis only if there is at least one valid bin in its proximity, *i*.*e*. the upstream and downstream 20 kb. BEDTools [60] were used to calculate the normalized read coverage in these bins at each time point. JTK_CYCLE [61] was applied to detect the circadian oscillation. We defined the minus logarithm of Bonferroni-adjusted p value of JTK_CYCLE, i.e. -log2(p), as the circadian index to measure circadian rhythmicity. To generate a meta-site for each binding factor, we computed the mean circadian index in each bin in the proximity of binding sites. The mock meta-site was obtained from randomly selected 100,000 sites of 40 kb in length over whole genome.

### Phase analysis of COGs from microarray data

The circadian time-series microarray data in mouse liver sampled every 1 hour for 48 hours were downloaded from GEO (accession: GSE11923) [5] to analyze the phases of COGs. We chose this time-series data for phase analysis because of its high temporal resolution. The raw data in CEL files were normalized by robust multi-array average (RMA). JTK_CYCLE was performed to obtain circadian phases at the probeset level on the microarray. R package mouse4302.db was used to annotate the gene symbols of 45,101 probesets. If one gene corresponds to multiple probesets, we only kept the one with the minimum Benjamini and Hochberg (BH) q value from JTK_CYCLE. 3,018 COGs were selected with the threshold of BH q value < 0.01. The genomic locations of these genes were obtained from UCSC genome (mm9 assembly).

To examine whether the neighboring COGs tend to have similar phases, we scanned the whole genome for COGs with neighboring double windows consisting of the upstream 20 kb and downstream 20 kb windows (S3C Fig). The phase differences were computed between two COGs situated in each of the double windows. If multiple COGs were found in one window, only the COG closest to the other window was retained. Next we increased the distance of two windows apart to 10, 20, 30, 40, and 50 kb and re-calculated the phase differences of COGs in the double windows. For a random genomic background, a pair of two 20 kb windows were randomly selected on the genome and searched for COGs. The phase differences were calculated for 1,000 such random pairs of windows. Compared to the strategy of just considering contiguous genes [62], our fixed-size window approach eliminates the distance factor between neighboring genes. Mann-Whitney U test was applied to detect the significance of difference in the distributions of phase difference between double windows and randomly chosen windows.

To obtain neighboring COGs separated by the binding sites of Bmal1, Nr1d1, Gabpa, cohesin-non-CTCF, and cohesin-CTCF, the transcription start sites of COGs were searched upstream 20 kb and downstream 20 kb relative to protein binding centers providing that the whole transcripts do not overlap with the binding centers. We selected the binding sites flanked by COGs and calculated the phase difference between the opposite sides of these binding sites. Mann-Whitney U test was applied to detect the significance of difference in the distributions of phase difference between across the binding factors and genomic background.

### Calculation of phase variance

The phase variance is calculated based on a method used to measure the dispersion of directional data [63]. In brief, the phase *p*_*i*_ of COG *i* is given by polar co-ordinates of unit length (cos *θ*_*i*_, sin *θ*_*i*_), *i* = 1,…,*n*. The mean of phases *p*_*0*_ is defined as the direction of the vector resulted from the vector summation $\left(\sum_{i=1}^{n}\frac{\text{cos}\;\theta_{i}}{\left|p_{0}\right|},\;\sum_{i=1}^{n}\frac{{sin\theta }_{i}}{\left|p_{0}\right|}\right)$, where $|p_{0}|=\sqrt{{(\sum_{i=1}^{n}\text{cos}\;\theta_{i})}^{2}+{\left(\sum_{i=1}^{n}\text{sin}\;\theta_{i}\right)}^{2}}$. The dispersion of phases is measured by $D(p_{0},p_{1})=\sum_{i=1}^{n}\left[1-\text{cos}(\theta_{0}-\theta_{i})]=n-\frac{{\left(\sum_{i=1}^{n}\text{cos}\;\theta_{i}\right)}^{2}+{\left(\sum_{i=1}^{n}\text{sin}\;\theta_{i}\right)}^{2}}{\left|p_{0}\right|}=n-|p_{0}\right|$. Hence, the phase variance is defined as 1 − |*p*_0_|/*n* after normalization by the sample size n. R package circular was used to calculate the phase variance.

We collected 23,724 intra-chromatin interactions from cohesin ChIA-PET data in mouse embryonic stem cells [29]. The invariant domains in mouse liver were inferred if two anchors of cohesin loops both overlapped with cohesin-CTCF binding sites in mouse liver. As a result, we obtained 16,837 invariant cohesin-CTCF domains. To explore the relationship between phase variance and window size, we scanned the whole genome with different sizes of windows 5×4^i^ kb, i = 1,2,…,5 to extract COGs and calculate the phase variance (S3D Fig). The Pearson’s correlation coefficient (PCC) was calculated between phase variance and log2 transformed window size. The p value for testing null hypothesis (PCC = 0) was computed based on Pearson’s product moment correlation coefficient. To reduce size effect in the comparison of phase variances between cohesin-CTCF domains and background, we classified cohesin-CTCF domains into small, medium, and large categories with sizes of [10×4^i^, 10×4^i+1^] kb (i = 1,2,3) respectively. The genomic background for each category is generated by the scan across genome with window of size 5×4^i+1^ (i = 1,2,3).

### A model of cohesin/CTCF dependent circadian gene regulation

We first defined a background model only considering the circadian regulation from nearby Bmal1 binding sites. In the background model, the regulatory potential *B*_*i*_ of Bmal1 on gene *i* is given by $B_{i}=\sum_{D_{\text{ij}}<2Mb}(e^{-D_{ij}/\lambda_{1}}\cdot S_{j})$, where *j* is Bmal1 binding site located within 2 Mb to gene *i*, *S*_*j*_ is the weight representing the signal of Bmal1 binding site *j* in ChIP-Seq data, and *D*_*ij*_ is the distance between gene *i* and Bmal1 binding site *j*. For cohesin/CTCF dependent model, the effects of cohesin-non-CTCF and cohesin-CTCF sites were multiplied upon the background model. For a given gene or Bmal1 site, we searched for the nearby cohesin-non-CTCF site within 5 kb that may facilitate gene regulation. We assigned a weight larger than 1 to the gene or Bmal1 binding site to increase the circadian regulatory potential. Between each pair of gene and Bmal1 binding site, we counted the number of cohesin-CTCF sites in between and assigned a weight less than 1 to reduce the circadian regulatory potential of Bmal1 on that gene. Taken together, the regulatory potential *P*_*i*_ of Bmal1 on gene *i* is given by $P_{i}=\sum_{D_{\text{ij}}<2Mb}\left[(e^{-\frac{D_{ij}}{\lambda_{1}}}\cdot S_{j})\cdot {CNC}_{i}\cdot {CNC}_{j}\cdot {CC}_{ij}\right]=\sum_{D_{\text{ij}}<2Mb}\left[(e^{-\frac{D_{ij}}{\lambda_{1}}}\cdot S_{j})\cdot (1+e^{-\frac{{ND}_{i}}{\lambda_{2}}}\cdot {SC}_{i})\cdot (1+e^{-\frac{{ND}_{j}}{\lambda_{2}}}\cdot {SC}_{j})\cdot {\left(\frac{1}{2}\right)}^{m_{ij}}\right]$, where *ND*_*i*_ and *ND*_*j*_ are the distances between the nearest cohesin-non-CTCF sites to gene *i* or Bmal1 site *j* respectively, *SC*_*i*_ and *SC*_*j*_ are the weights representing their signals on cohesin ChIP-Seq data, and *m*_*ij*_ is the number of cohesin-CTCF sites between gene *i* and Bmal1 *j*. If there is no cohesin-non-CTCF within 5 kb of gene *i* or Bmal1 site *j*, $e^{-\frac{{ND}_{i}}{\lambda_{2}}}$ or $e^{-\frac{{ND}_{j}}{\lambda_{2}}}$ was assigned to 0. The weights *S* and *SC* are defined by $1+e^{-\frac{r}{\lambda_{3}}}$, where *r* is the rank of ChIP signal among all Bmal1 or cohesin binding sites respectively. The parameters *λ*_*1*_, *λ*_*2*_, *λ*_*3*_ are set to be 2000000/4, 5000/4, and (total number of peaks in ChIP-Seq)/4 respectively as suggested by an empirical model of gene regulation [64]. To render the circadian regulatory potentials directly comparable between two models, we finally converted them to their respective ranks in the models as Rank(*P*_*i*_)/*n* where *n* is the total number of genes considered.

### *Smc3*-/- mouse embryonic fibroblast cells

*Smc3*-flox/flox MEF (mouse embryonic fibroblast) cells was originally derived from European conditional mouse mutagenesis program [65] (http://www.informatics.jax.org/allele/MGI:4434007). The Cre/GFP adenovirus and GFP adenovirus (10^10^ pfu/ml) were purchased from Hanbio biotechnology, Shanghai. MEF cells were cultured with 10% FBS in DMEM (Life technology). To avoid the loss of viability in *Smc3*-/- cells when they enter mitosis, we infected the cells at G0/1 stage of the cell cycle. The medium was changed two days after the cells reaching the complete confluence. 10^9^ pfu GFP and Cre/GFP adenovirus were used in 8-hr treatment for wild type and *Smc3*-/- MEF cells respectively. To allow the cells to recover from viral infection, we changed the medium into serum-free DMEM and kept the cells for 6 days at high confluence. MEF cells were then synchronized by dexamethasone (Sigma) with the final concentration of 100 nM for 1 hr. The cells were rinsed with PBS and cultured with serum-free DMEM. Wild type and *Smc3*-/- MEF cells were collected at 20, 24, 28, 32, 36, and 40 hr after synchronization. Total RNA was extracted using Trizol reagent and reverse-transcribed into cDNA by SuperScript II RT (Life Technologies). RNA-Seq libraries for 20 hr and 32 hr samples were prepared by using Illumina TruSeq RNA Sample Prep Kit V2 and were subjected to deep sequencing with 1×100 bp read on HiSeq 2000 at CAS-MPG Partner Institute for Computational Biology Omics Core, Shanghai, China (S6 Table). RNA-Seq reads were mapped to mouse reference genome (mm9 assembly) by Tophat [66]. HTSeq was used to count the number of uniquely mapped reads that are located on the exons of genes [67]. Only genes with at least one read in all samples were kept for downstream analysis. Treating 20 hr and 32 hr samples as biological replicates, we applied DESeq to select differentially expressed genes between cohesin knockout and control cells with log2 fold change > 0.8 [68]. The Gene Expression Omnibus (GEO) accession number for RNA-Seq dataset is GSE68831.

Bmal1 ChIP in MEF cells were performed following the protocol by Shimomura et al. [69] with modification. Briefly, 10^7^ cells were washed with PBS and cross-linked by 1% formaldehyde for 10 min on a rocker at room temperature. The cross-linking was quenched by 2.5 M Glycine with final concentration of 125 mM. The nuclei was extracted at 4°C from the homogenate by lysis buffer containing protease inhibitors [50mM Hepes-KOH, pH 7.5, 140mM NaCl, 1mM EDTA, 10% glycerol, 0.5% NP-40, 0.25% Triton X-100], [10mM Tris-HCl, pH 8.0, 200mM NaCl, 1mM EDTA, 0.5 mM EGTA], and [10mM Tris-HCl, pH 8.0, 200mM NaCl, 1mM EDTA, 0.5 mM EGTA, 0.1% Na-Deoxycholate, 0.5% N-lauroylsarcosine]. DNA was fragmented with sonication into 150–300 bp at 4°C. 50 μl DNA fragments were stored in 4°C as the input DNA. The rest of DNA fragments were incubated on rocker at 4°C for 6 hr with 1:1 ChIP buffer [20% Triton, NaDOC, NaCl, TE, inhibitor] and 4 μl Bmal1 antibody (Santa Cruz: sc-8550). Then 15 μl protein A/G agarose beads were added into DNA and incubated on rocker at 4°C overnight. Co-immunoprecipitated DNA was washed with 1 ml buffers [5% Triton, 1% SDS, 1% NaDOC, 93% TE] twice, [5% Triton, 1% SDS, 1% NaDOC, 6% NaCl, 87% TE] twice, [10% LiCl, 5% NP40, 5% Na-DOC, 80% TE] twice, [10% Triton, 90% TE], and TE. Then DNA was reverse cross-linked at 50°C for 2 hr with TE 100 μl, 10% SDS 3 μl, and protease K 5 μl. QIAquick PCR Purification Kit (QIAGEN) was used to purify ChIP DNA. Input and ChIP DNA library were prepared by using Illumina TruSeq ChIP Sample Prep Kit and were subject to deep sequencing with 1×100 bp read on HiSeq 2000 at CAS-MPG Partner Institute for Computational Biology Omics Core, Shanghai, China (S6 Table). ChIP-Seq data analysis was performed in the same pipeline described above. The Gene Expression Omnibus (GEO) accession number for Bmal1 ChIP-Seq data set is GSE77162.

### The genome editing of CRISPR-Cas9 system

CRISPR-Cas9 method [70] was used to delete the cohesin binding site near the enhancer of *Phldb2* in Hepa1-6 cells. The gRNA target sequences (GTCTTTCACGTGGGACGGAT and GAGACCTCAAGGACATGTGC) were designed by E-CRISP [71]. The homologous arms for donor plasmids are (chr16: 45967525–45967702) and (chr16: 45967935–45968129). The regulatory module (hPGK promoter/PuroR) was amplified from commercially available expression vector pLKO.1. Two homologous arms and PGK/puroR were assembled into pGEM-T Easy vector (Promega). Hepa1-6 cells were cultured with 10% FBS in DMEM (Life technology) and co-transfected with two gRNA/Cas9 vectors and linearized donor DNA. Then the cells were screened with 3 μg/ml puromycin (Merck/ millipore) for 2 weeks. Gel electrophoresis analysis of the homologous arms, control region, and the regulatory module (PGK-puroR) in WT and CRISPR-CAS9 treated cells validated the successful deletion of target DNA region (S6B Fig). Primers used in PCR and RT-PCR are listed in S5 Table.

The whole-genome scans in this study were implemented in Java language (JDK 6). All statistical analyses were performed in R 2.11.

## Supporting Information

S1 Fig (a) The Bmal1-bound circadian enhancer upstream 8 kb of *Nr1d1*. The genome browser shows the binding profiles of H3K4me1, H3K27ac, Bmal1 (in liver and MEFs), Nr1d1, cohesin, and CTCF around the 4C bait. Bmal1 ChIP-Seq in MEFs was conducted in this study and other ChIP-Seq data showed in here were re-analyzed from published data (Methods). (b) The Bmal1 enhancers ranked by Bmal1 signal on ChIP-Seq in mouse liver (GSE39860, GSE26602). The top 3% rank of enhancers were defined as Bmal1 super-enhancers (Methods). The enhancer selected as 4C bait has the highest Bmal1 signal. (c) The cumulative curves of 4C reads on the cis-chromosome of the enhancer. In all samples of CT16 and CT18, over 40% reads are mapped to 150 kb region around the bait.
(TIFF)

S2 Fig The read profiles of 4C samples in the upstream and downstream 2 Mb region to the bait. The highly interacting region (150 kb to the bait) is indicated by the region between two red lines. The Hi-C data in mouse embryonic stem cells [12] showed that highly interacting region of the enhancer is restricted in a topologically associating domain (black box).
(TIFF)

S3 Fig (a) The circadian index around protein binding sites defined from the transcription of positive strand in GRO-Seq (GSE59486, Methods). The result from negative strand was showed in Fig 3A. (b) The circadian index around protein binding sites defined from the transcription in RNA-Seq (GSE39860, Methods). (c) A depiction of our procedure to calculate the phase differences of COGs across a given protein binding site, genome background, and random background. The black arrow represents a scan across the genome. The gene phases were calculated based on time-profiling microarray (GSE11923). The violin plot shows the genome background of phase differences of COGs in 20–20 kb double windows. The interval sizes between the two windows are indicated at the bottom. In the case of random background, a pair of two 20-kb windows were randomly selected on the genome. The asterisks indicate Mann-Whitney U test p value compared to random background. (d) A depiction of our procedure to calculate the phase variance from inferred domains and genome background. The violin plot shows the genome background of phase variances of COGs in different sizes of windows, which is indicated below. The phase variance is positively correlated to log2-transformed window size. ****p < 10^−8^, ***p < 10^−4^, **p < 0.01, *p < 0.05.
(TIFF)

S4 Fig (a) The distributions of Nr1d1 regulatory potentials for COGs and non-COGs in background model and cohesin/CTCF dependent model. The COGs in cohesin/CTCF dependent model as well as in the model with only cohesin-CTCF or cohesin-non-CTCF effect have significantly higher potentials than in background model (KS test, p = 10^−16^). (b) The distribution of the phases of COGs with top 10% Nr1d1 regulatory potentials in cohesin/CTCF dependent model. The phase of Nr1d1 binding (CT10) is indicated by dash line. (c) The scatterplots of regulatory potentials in model and background for 20 clock genes. The color bar indicates the phases of clock genes.
(TIFF)

S5 Fig (a) The genome browser shows the binding profiles of cohesin, Nr1d1, H3K4me1, H3K27ac, cohesin loop around *Ahnak*, and RNA-Seq profile in *Smc3*-/- MEFs. The locations of the 3C primers for enhancer-promoter interaction analysis were indicated. Two Nr1d1 binding sites (marked by asterisk) are located downstream 106 kb and 108 kb of *Ahnak* respectively. (b) The 3C signals of interactions anchored to *Ahnak* promoter in mouse liver (ANOVA, p = 10^−9^, mean+/-SD, 2 biological replicates, 4 technical replicates). The positions of primers were indicated in (a). CN, control. EN, enhancer. (c) The normalized 3C signals between the promoters of *Ahnak* and its enhancer in control and *Smc3*-/- MEFs (Student’s t-test, p = 0.009, mean+/-SD, 2 biological replicates, 3 technical replicates).
(TIFF)

S6 Fig (a) The Bmal1 ChIP-qPCR signal relative to input on the enhancer of *Phldb2* in mouse liver (compared to IgG ChIP t-test p = 0.001, mean+/-SD, 2 biological replicates, 2 technical replicates). (b) CRISPR-CAS9 deletion of the cohesin binding site near the enhancer of *Phldb2* in Hepa1-6 cells. Gel electrophoresis shows the homologous arms (Homology), control region (Con), and the regulatory module (PGK-puroR) in WT and CRISPR-CAS9 treated cells (Methods).
(TIFF)

S1 Table Ninety-seven Bmal1super-enhancers in mouse liver ranked by Bmal1 signal. (XLSX)

S2 Table Enhancer interacting regions at CT6 and CT18. (XLSX)

S3 Table Bmal1 regulatory potentials in cohesin/CTCF dependent model and background model. The 3rd and 4th columns show the regulatory potentials when we only incorporate cohesin-non-CTCF or cohesin-CTCF effect in background model. The JTK_cycle q value and circadian phases of genes in mouse liver are showed in 6th and 7th column respectively. In this study, we consider q < 0.01 as the criteria of COGs.
(XLSX)

S4 Table Peak calling results for Bmal1 ChIP-Seq in MEF cells and DESeq results for RNA-Seq in control and Smc3-/- MEFs. (XLSX)

S5 Table All primers used in this study. (XLSX)

S6 Table The statistics of sequencing reads of 4C-Seq, ChIP-Seq, and RNA-Seq conducted in this study. (XLSX)
