# Supplementary figures and images for: Long-Range Chromosome Interactions Mediated by Cohesin Shape Circadian Gene Expression
Source: PLoS Genet. 2016 May 2;12(5):e1005992. doi: 10.1371/journal.pgen.1005992 (PMC4852938; doi:10.1371/journal.pgen.1005992)

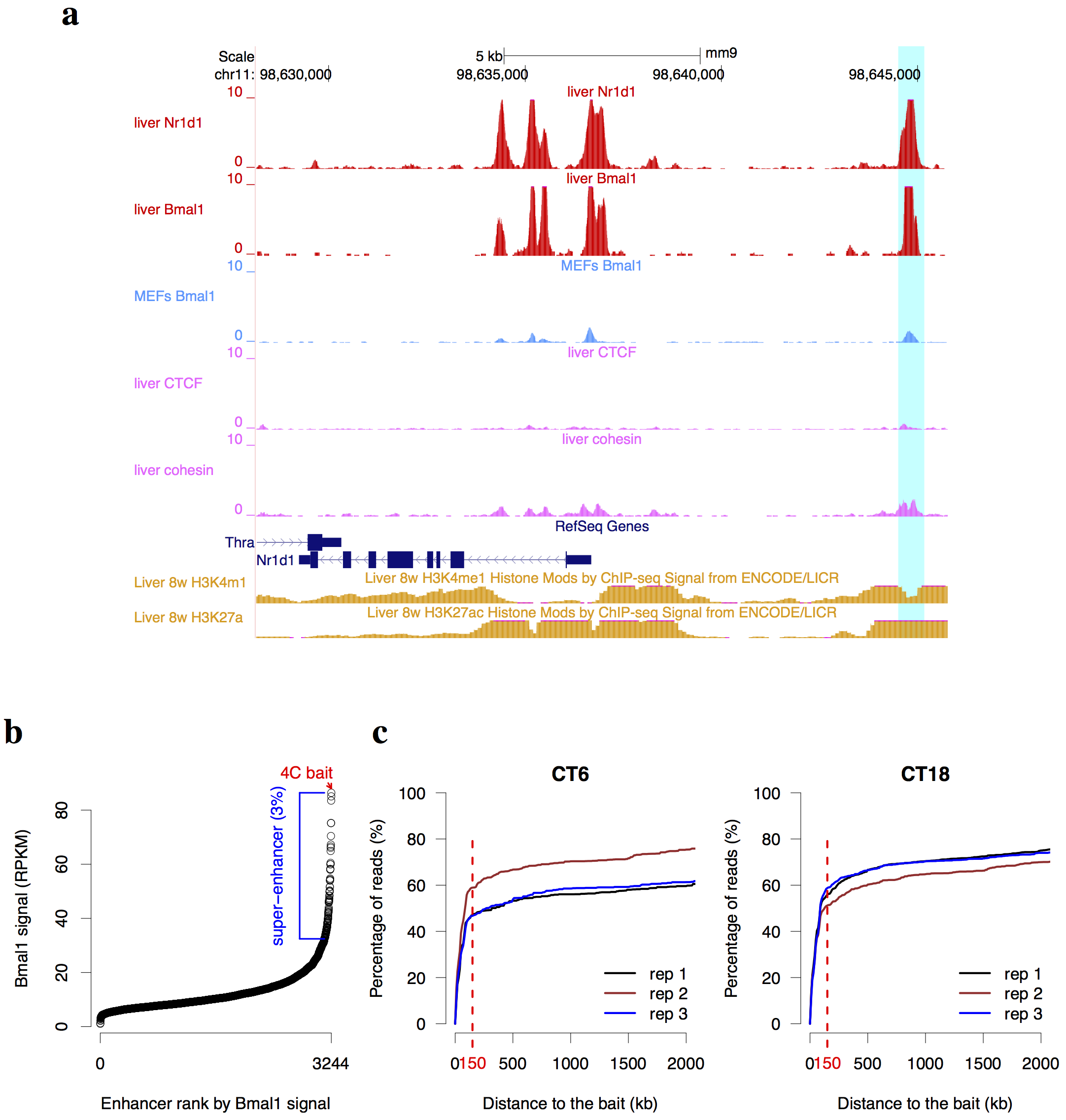

Supplement: S1 Fig — (a) The Bmal1-bound circadian enhancer upstream 8 kb of Nr1d1. The genome browser shows the binding profiles of H3K4me1, H3K27ac, Bmal1 (in liver and MEFs), Nr1d1, cohesin, and CTCF around the 4C bait. Bmal1 ChIP-Seq in MEFs was conducted in this study and other ChIP-Seq data showed in here were re-analyzed from published data (Methods). (b) The Bmal1 enhancers ranked by Bmal1 signal on ChIP-Seq in mouse liver (GSE39860, GSE26602). The top 3% rank of enhancers were defined as Bmal1 super-enhancers (Methods). The enhancer selected as 4C bait has the highest Bmal1 signal. (c) The cumulative curves of 4C reads on the cis-chromosome of the enhancer. In all samples of CT16 and CT18, over 40% reads are mapped to 150 kb region around the bait. (TIFF) [file pgen.1005992.s001.tiff]

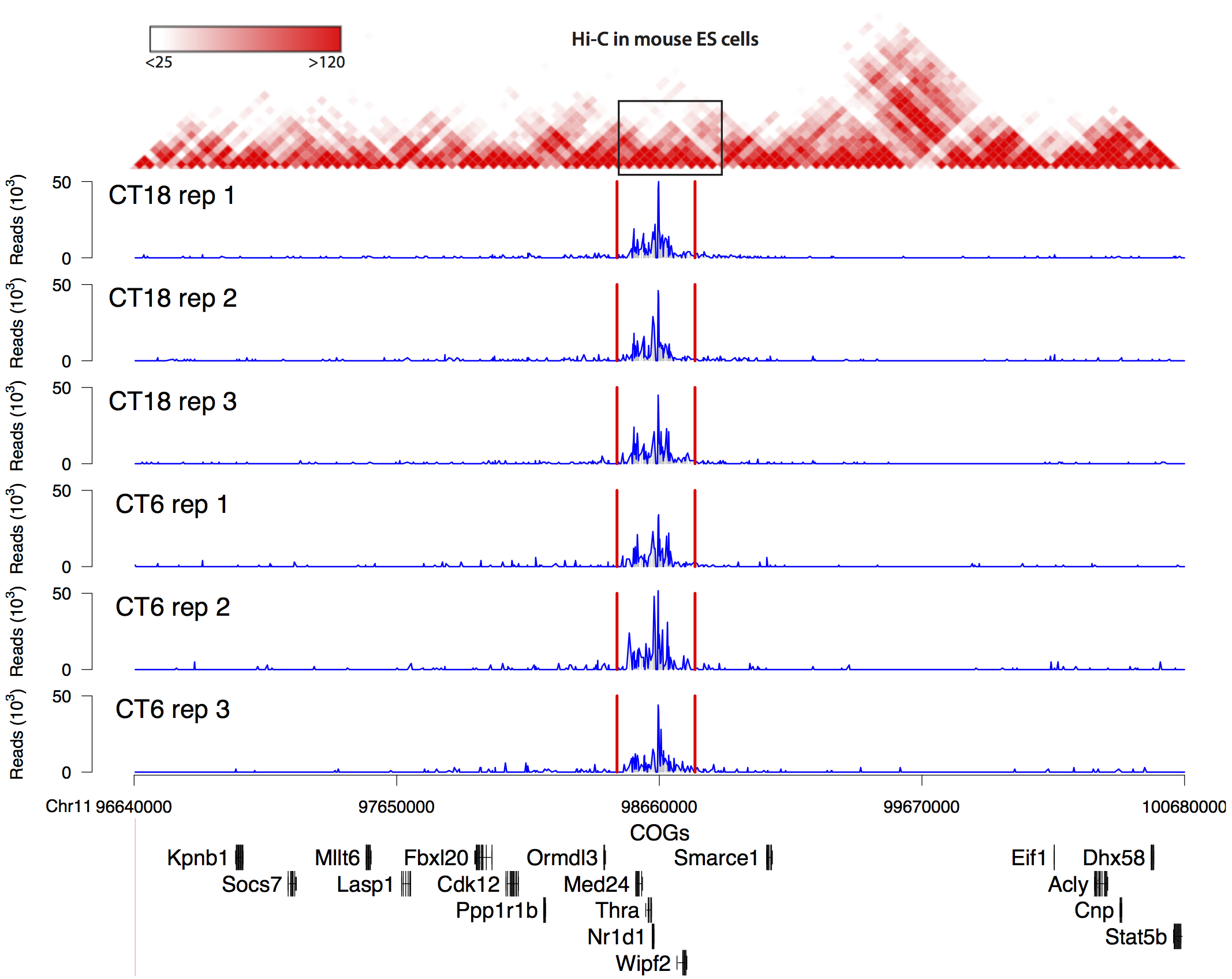

Supplement: S2 Fig — The highly interacting region (150 kb to the bait) is indicated by the region between two red lines. The Hi-C data in mouse embryonic stem cells [12] showed that highly interacting region of the enhancer is restricted in a topologically associating domain (black box). (TIFF) [file pgen.1005992.s002.tiff]

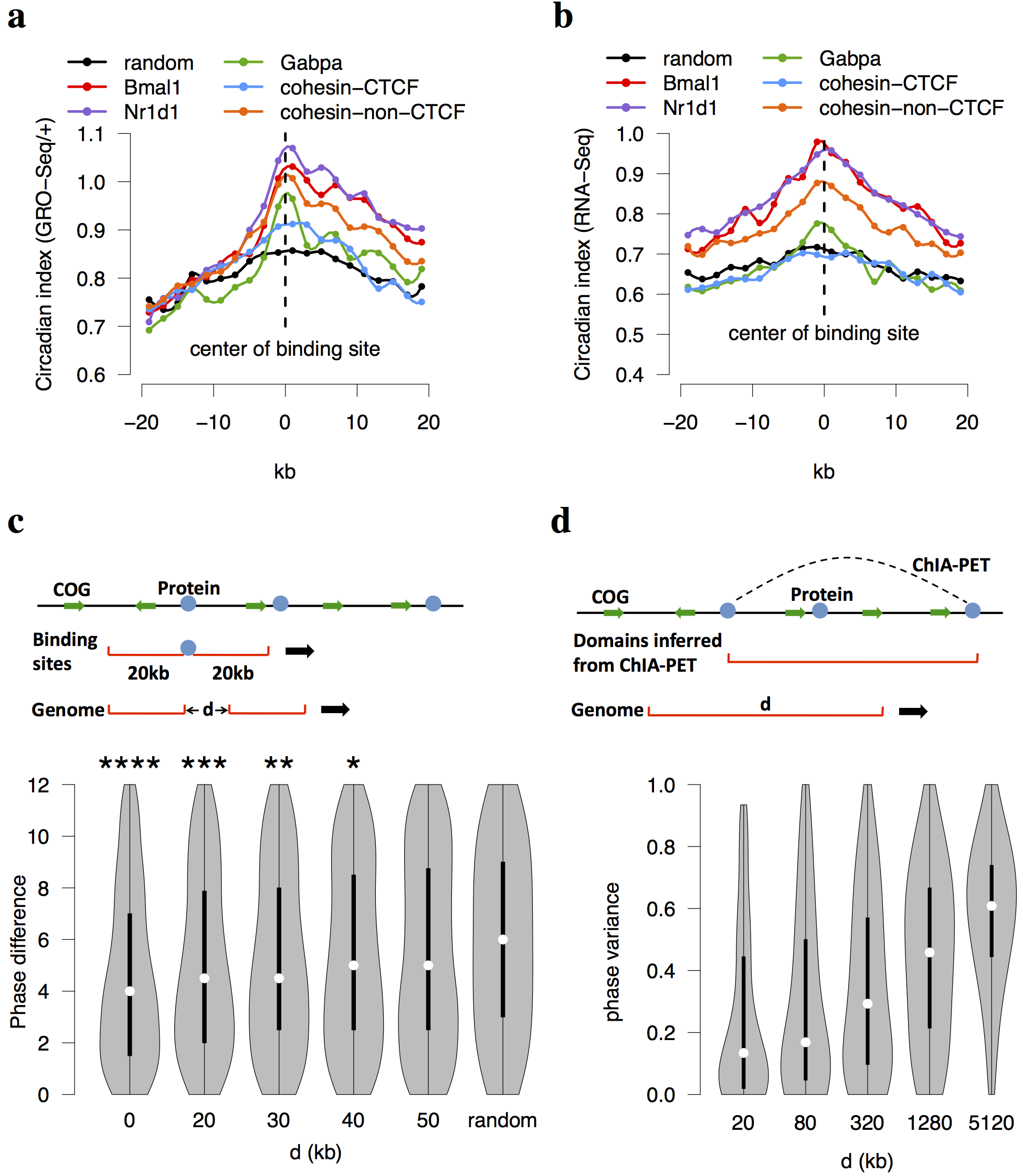

Supplement: S3 Fig — (a) The circadian index around protein binding sites defined from the transcription of positive strand in GRO-Seq (GSE59486, Methods). The result from negative strand was showed in Fig 3A. (b) The circadian index around protein binding sites defined from the transcription in RNA-Seq (GSE39860, Methods). (c) A depiction of our procedure to calculate the phase differences of COGs across a given protein binding site, genome background, and random background. The black arrow represents a scan across the genome. The gene phases were calculated based on time-profiling microarray (GSE11923). The violin plot shows the genome background of phase differences of COGs in 20–20 kb double windows. The interval sizes between the two windows are indicated at the bottom. In the case of random background, a pair of two 20-kb windows were randomly selected on the genome. The asterisks indicate Mann-Whitney U test p value compared to random background. (d) A depiction of our procedure to calculate the phase variance from inferred domains and genome background. The violin plot shows the genome background of phase variances of COGs in different sizes of windows, which is indicated below. The phase variance is positively correlated to log2-transformed window size. ****p < 10−8, ***p < 10−4, **p < 0.01, *p < 0.05. (TIFF) [file pgen.1005992.s003.tiff]

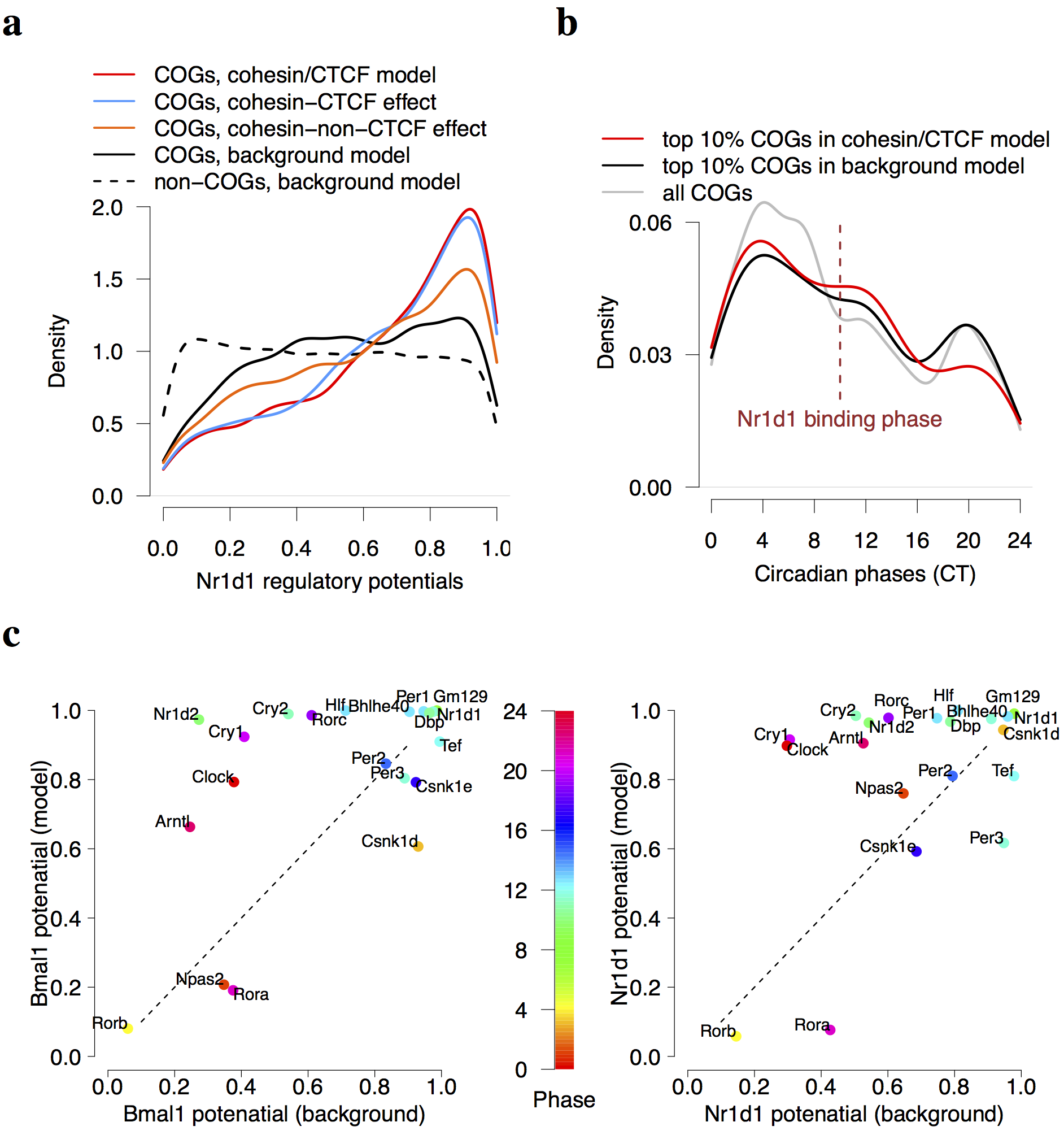

Supplement: S4 Fig — (a) The distributions of Nr1d1 regulatory potentials for COGs and non-COGs in background model and cohesin/CTCF dependent model. The COGs in cohesin/CTCF dependent model as well as in the model with only cohesin-CTCF or cohesin-non-CTCF effect have significantly higher potentials than in background model (KS test, p = 10−16). (b) The distribution of the phases of COGs with top 10% Nr1d1 regulatory potentials in cohesin/CTCF dependent model. The phase of Nr1d1 binding (CT10) is indicated by dash line. (c) The scatterplots of regulatory potentials in model and background for 20 clock genes. The color bar indicates the phases of clock genes. (TIFF) [file pgen.1005992.s004.tiff]

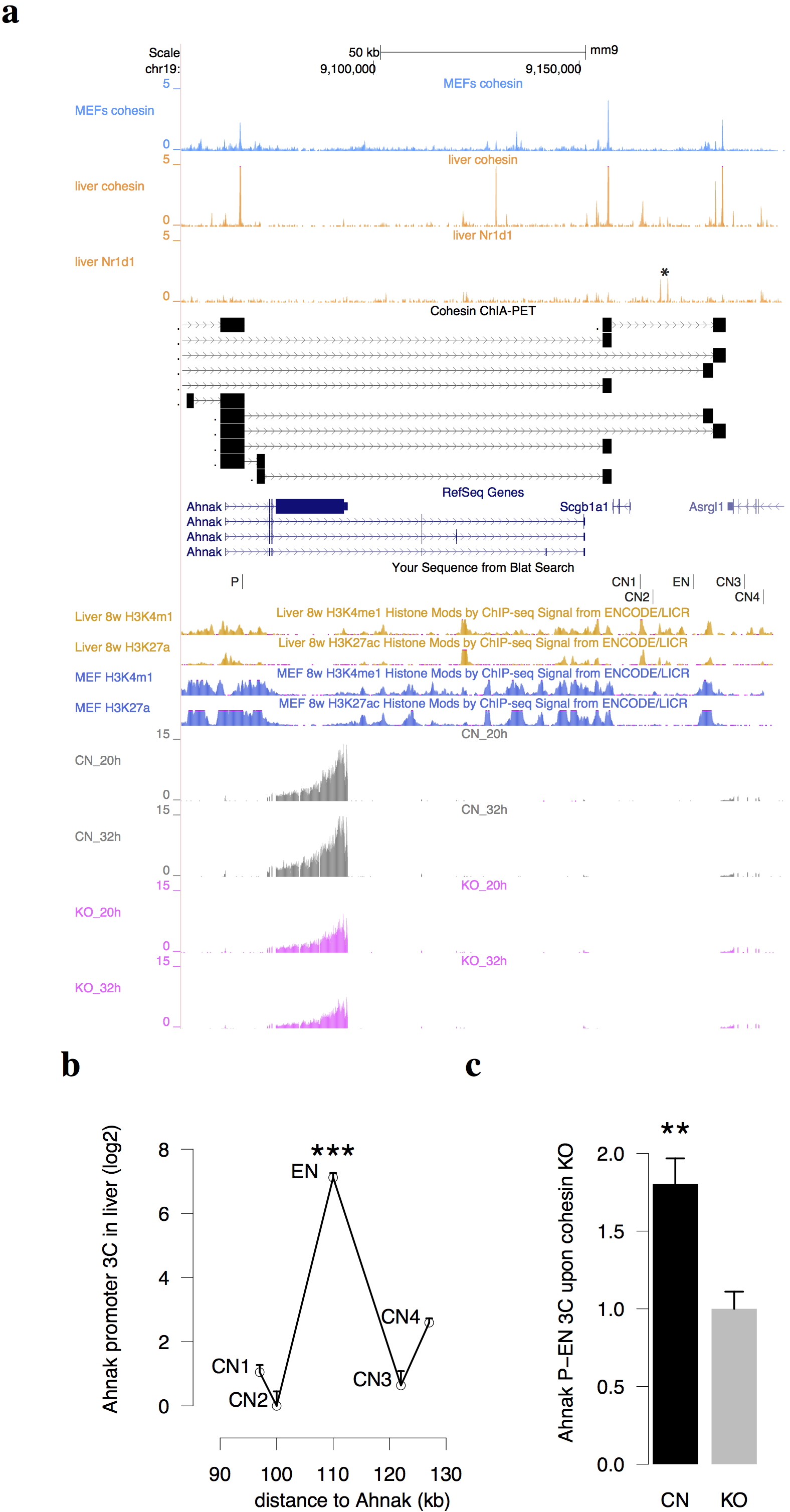

Supplement: S5 Fig — (a) The genome browser shows the binding profiles of cohesin, Nr1d1, H3K4me1, H3K27ac, cohesin loop around Ahnak, and RNA-Seq profile in Smc3-/- MEFs. The locations of the 3C primers for enhancer-promoter interaction analysis were indicated. Two Nr1d1 binding sites (marked by asterisk) are located downstream 106 kb and 108 kb of Ahnak respectively. (b) The 3C signals of interactions anchored to Ahnak promoter in mouse liver (ANOVA, p = 10−9, mean+/-SD, 2 biological replicates, 4 technical replicates). The positions of primers were indicated in (a). CN, control. EN, enhancer. (c) The normalized 3C signals between the promoters of Ahnak and its enhancer in control and Smc3-/- MEFs (Student’s t-test, p = 0.009, mean+/-SD, 2 biological replicates, 3 technical replicates). (TIFF) [file pgen.1005992.s005.tiff]

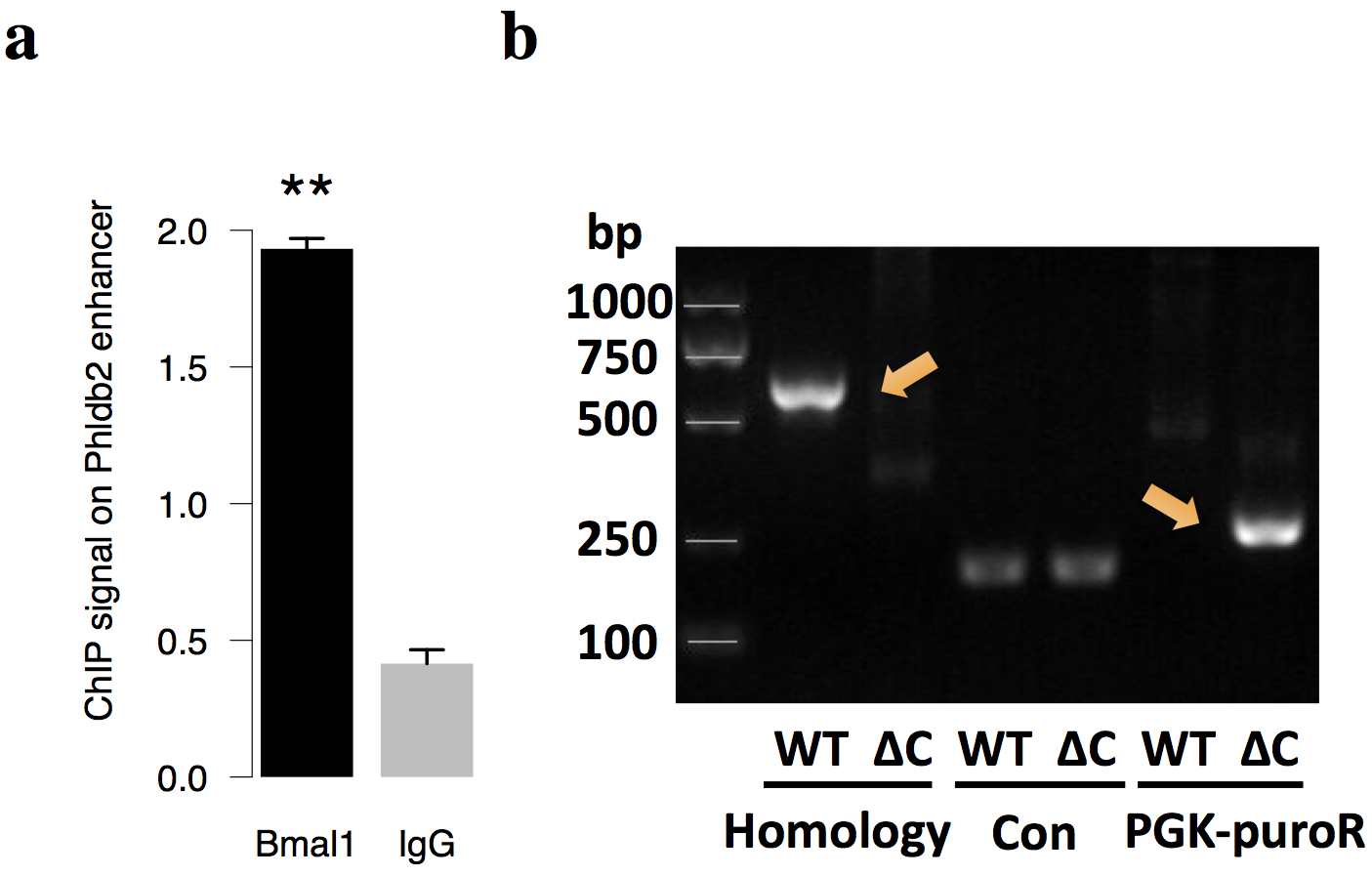

Supplement: S6 Fig — (a) The Bmal1 ChIP-qPCR signal relative to input on the enhancer of Phldb2 in mouse liver (compared to IgG ChIP t-test p = 0.001, mean+/-SD, 2 biological replicates, 2 technical replicates). (b) CRISPR-CAS9 deletion of the cohesin binding site near the enhancer of Phldb2 in Hepa1-6 cells. Gel electrophoresis shows the homologous arms (Homology), control region (Con), and the regulatory module (PGK-puroR) in WT and CRISPR-CAS9 treated cells (Methods). (TIFF) [file pgen.1005992.s006.tiff]
